# Nuclear localization of BRCA1-associated protein 1 is important in suppressing hepatocellular carcinoma metastasis via CTCF and NRF1/OGT axis

**DOI:** 10.1038/s41419-025-07451-0

**Published:** 2025-02-21

**Authors:** Xiaoyu Xie, Yu-Man Tsui, Vanilla Xin Zhang, Tiffany Ching-Yun Yu, Abdullah Husain, Yung-Tuen Chiu, Lu Tian, Eva Lee, Joyce Man-Fong Lee, Hoi-Tang Ma, Daniel Wai-Hung Ho, Karen Man-Fong Sze, Irene Oi-Lin Ng

**Affiliations:** 1 Department of Pathology, The University of Hong Kong, Pokfulam, Hong Kong; 2 State Key Laboratory of Liver Research, The University of Hong Kong, Pokfulam, Hong Kong

**Keywords:** Liver cancer, Oncogenesis

## Abstract

Germline mutations of the deubiquitinase BRCA1-associated protein 1 (BAP1) lead to the “BAP1 cancer syndrome” characterized by development of cancers. However, the role of BAP1 in hepatocellular carcinoma (HCC) is unclear. We found that BAP1 was upregulated at mRNA level in human HCCs and significantly correlated with a more aggressive tumour behaviour. Intriguingly, we observed cytoplasmic but no or minimal nuclear BAP1 in human HCC samples by immunohistochemistry. We observed that, while BAP1 protein was found mainly in the cytoplasm and less in the nuclei of HCC cell lines, BAP1 expression was predominantly nuclear in HepG2 cells, by cell fractionation and immunofluorescence analyses. Functionally, in the orthotopic liver injection mouse model, silencing the BAP1 predominant nuclear expression of HepG2 cells promoted intrahepatic tumor metastasis, with more frequent tumor microsatellite formation and venous invasion. With transcriptomic profiling, we identified RHOJ amongst the downregulated targets in HepG2 cells upon BAP1 knockdown. Subsequent overexpression of RHOJ suppressed cell migration in HCC cells, suggesting that BAP1 might upregulate RHOJ resulting in reduced cell migratory ability of HCC cells. Furthermore, we identified two transcription factors, CTCF and NRF1, which activated BAP1 transcription by binding to BAP1 promoter region. On the other hand, we uncovered that O-linked N-acetylglucosamine (GlcNAc) transferase (OGT) physically bound to BAP1 in the nucleus, resulting in diminished stability of the nuclear BAP1. Intriguingly, OGT transcription was upregulated and was also under the control of CTCF and NRF1 in human HCC, acting as a negative regulator of BAP1. To summarize, this study uncovered the underlying mechanisms of the regulation of BAP1 and that loss of the nuclear localization of BAP1 protein contributed to enhanced cell migration in vitro and more aggressive tumor behavior in human HCCs.

## Introduction

Hepatocellular carcinoma (HCC) is prevalent malignancy worldwide and ranks as the sixth most common and third most fatal in cancer mortality [1, 2]. However the molecular mechanisms of hepatocarcinogenesis are varied [3]. Deubiquitinating enzymes are a group of enzymes that cleave ubiquitin from ubiquitinated proteins, hence promoting their protein stability as well as cell signaling cascades. Until now, there are more than 100 genes encoding deubiquitinating enzymes in humans. BRCA1-associated protein 1 (BAP1), encoded by the BAP1 gene, belongs to the ubiquitin C-terminal hydrolase (UCH) superfamily, first identified as a BRCA1 Ring finger domain binding protein by yeast two-hybrid screen in 1998. BAP1 is located on chromosome 3p21.3 [4].

Human BAP1 protein consists of a total of 729 amino acids and possesses a UCH domain at its N-terminus, indicating that BAP1 is a thiol-dependent deubiquitinase (DUB) [4]. Unlike other UCH family proteins, BAP1 has a long C-terminal extension providing many binding sites for its interacting proteins such as OGT, FOXK1 and 2, KDM1B, YY1, and HCF1 [5]. Different complexes containing BAP1 may form, based on different cellular environments and consequently may determine some tissue-dependent gene transcriptions [6, 7]. BAP1 is positively involved in the regulation of chromatin and also binds to some transcription factors and cofactors, thereby bridging chromatin-remodeling complexes and chromatin [8, 9]. By means of deubiquitinating HCF1, which actively participates in the process of transcription, BAP1 is recognized as a cell proliferation regulator [8, 10]. Interacting with the transcription factor FOXK2, BAP1 is similarly recruited to DNA, thus deubiquitinating local histones and regulating the activity of the target genes [11]. In addition, two nuclear localization signal (NLS) domains are present at the C-terminus of BAP1, implicating that localization at the nucleus is important for BAP1 to exert its functions. Indeed, it has been reported that nuclear localization is required for its tumor suppressor function [12].

BAP1 has been reported to be a tumor suppressor in many cancers, for instance, uveal melanoma and mesothelioma, while conversely it has been reported to promote cell proliferation in esophageal carcinoma [13]. Thus, the role of BAP1 in cancer development is controversial. In HCC, the role of BAP1 is unclear. In this study, we aimed to investigate BAP1 in HCC from different aspects, including its expression, subcellular localization, functions, and transcription regulation.

## Material and methods

The details of materials and methods used in this study are described in Supplementary information. The original data of western blots are reported as Original Data 1.

## Results

### Upregulation of BAP1 mRNA expression in human HCCs

**Fig. 1:**
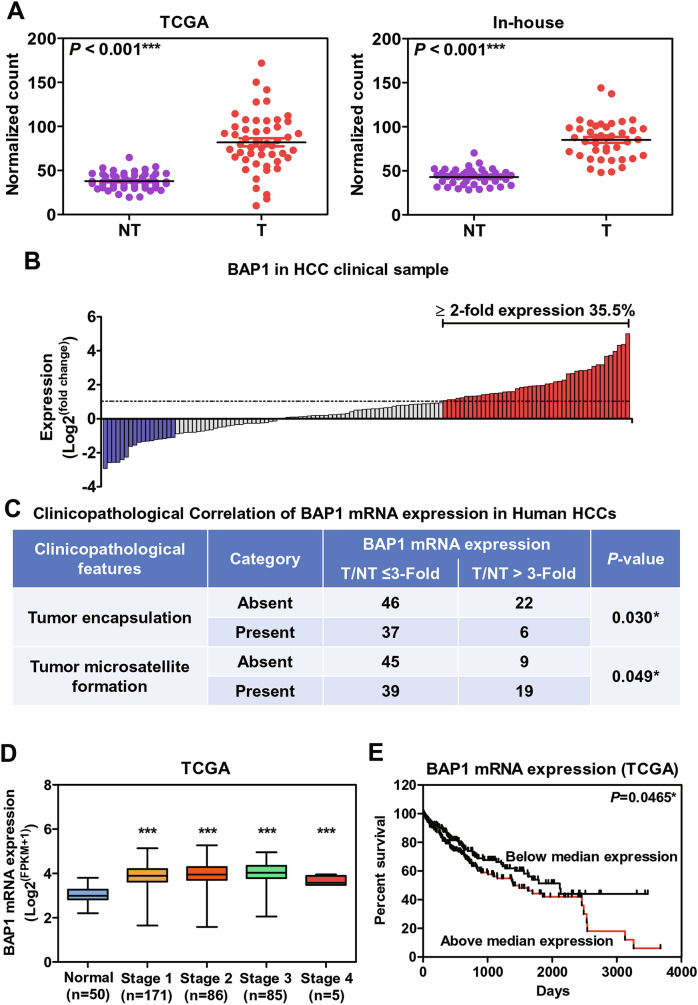
BAP1 mRNA level was upregulated in human HCC with more aggressive tumor behavior. **A** BAP1 mRNA expression in HCC tumor tissues and non-tumorous liver tissues in TCGA-LIHC (*n* = 50) and our in-house HCC (*n* = 41) cohorts. T: Tumor tissue, NT: Non-tumorous liver tissue. (***P < 0.001, Student’s t-test). **B** BAP1 mRNA expression in tumors and corresponding non-tumorous livers by qRT-PCR in in-house HCC cohort (*n* = 124). 35.5% of the tumors showed ≥2-fold upregulation. **C** Clinicopathological correlation of BAP1 mRNA expression in human HCCs. **D** Upregulation of BAP1 mRNA expression in different stages of HCC. (***P < 0.001, Student’s t-test) **E** Overall survival analysis of HCC patients with high or low BAP1 expression in TCGA-LIHC cohort. (*P < 0.05, Log rank test).

From TCGA-LIHC and our in-house RNA-sequencing cohorts, BAP1 was significantly upregulated at mRNA level in both (Fig. 1A) (*P* < 0.0001 for both). We validated the upregulation of BAP1 mRNA levels in an independent University of Hong Kong-Queen Mary Hospital (HKU-QMH) cohort comprising 124 HCC cases using qRT-PCR. There was significant upregulation of BAP1 in HCC as compared with the corresponding non-tumorous livers (*P* < 0.0001), with 35.5% (*n* = 44/124) of the cases showed more than 2-fold upregulation (Fig. 1B). Upon clinicopathological analysis, overexpression of BAP1 at mRNA level was significantly correlated with a more aggressive tumor behavior, with absence of tumor encapsulation (*P* = 0.030) and presence of tumor microsatellite formation (*P* = 0.049), which is a feature of intrahepatic metastasis (Fig. 1C). In addition, BAP1 mRNA was upregulated from the early stages to late stages of HCC progression, as compared with normal subjects (Fig. 1D). Its upregulation was also associated with poor overall survival (*P* = 0.0465) in HCC patients (Fig. 1E). These observations somewhat contradicted the general notion that BAP1 is a tumor suppressor, and further research into the role of BAP1 in HCC was justified.

### Overexpression of cytoplasmic BAP1 protein in human HCCs

**Fig. 2:**
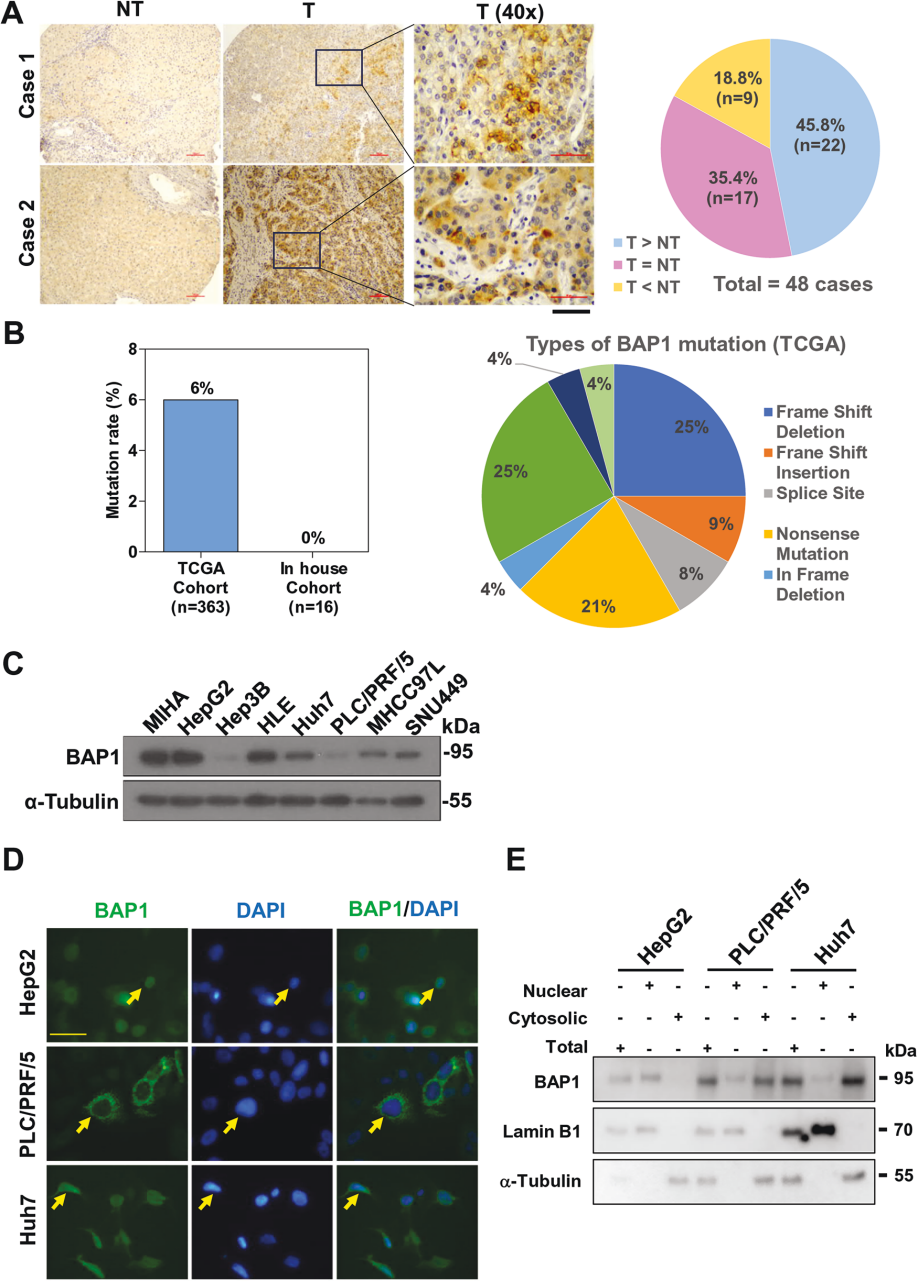
Cytoplasmic BAP1 was upregulated in human HCCs. **A** Representative immunohistochemical staining of BAP1 protein on TMAs of human HCCs (upper panel) Black scale bar, 50 μm. The pie chart represents the ratios of different groups of BAP1 protein expression as detected by immunohistochemistry on the TMAs from HCC clinical samples (lower panel). T: Tumor tissue, NT: Non-tumorous liver tissue. Scale bar, 100 μm. **B** BAP1 mutation rate detected by RNA sequencing in TCGA-LIHC cohort and our in-house HCC cohort by whole exome sequencing. The pie chart represents the ratios of different BAP1 mutation types in TCGA-LIHC cohort. **C** Western blot showing the protein expression levels of HCC cell lines. **D** Immunofluorescence staining showed that BAP1 localized mainly in the nuclei of HepG2 cells and in both nuclei and cytoplasm of PLC/PRF/5 and Huh7 cells. Scale bar, 50 μm. **E** Subcellular fractionation confirmed the predominant nuclear localization of BAP1 in HepG2 cells and predominant cytoplasmic localization in PLC/PRF/5 and Huh7 cells. α-Tubulin served as internal control of cytoplasmic protein fraction while Lamin B1 served as internal control of nuclear protein fraction. The total protein of each cell line served as loading control before cell fractionation.

To examine the protein expression and sub-cellular localization of BAP1, we conducted immunohistochemistry using tissue microarray (TMA) on a total of 48 pairs of HCC and corresponding non-tumorous liver samples from our patients’ resected samples at HKU-QMH, Hong Kong. Twenty-two (45.83%) of the 48 cases showed a higher expression in the HCC tumors than their corresponding non-tumorous liver tissues (Fig. 2A); 17 (35.43%) cases had comparable expression of BAP1 between the two; and 9 (18.75%) cases had lower BAP1 expression in HCCs than the corresponding non-tumorous livers. To our surprise, the BAP1 protein was detected only in the cytoplasm of tumor cells in patients’ HCCs, and no clear nuclear stain was detected in the nuclei (Fig. 2A). Since mutations at C-terminus of BAP1 can lead to loss of NLS, to rule out this possibility, we examined the mutations in BAP1 gene. The BAP1 mutation rates in TCGA-LIHC cohort and our in-house whole exome-sequencing in 16 HCCs were low, with 6% and 0%, respectively (Fig. 2B).

In general, BAP1 was ubiquitously expressed at protein levels in HCC cell lines as well as in immortalized liver cell line MIHA (Fig. 2C). Furthermore, we observed that with immunofluorescence staining, BAP1 was localized predominantly in the nuclei of HepG2 cells but localized in both nuclear and cytoplasm in PLC/PRF/5 and Huh7 cells (Fig. 2D). Similarly, with cell fractionation, BAP1 was mainly found in the nuclear fraction whereas it was found predominantly in the cytoplasmic fraction in both PLC/PRF/5 and Huh7 cells (Fig. 2E). Taken altogether, these findings showed that BAP1 was upregulated at the mRNA and protein levels in human HCC tumors and was independent of BAP1 mutations in the tumors. It was localized in different compartments of HCC cell lines.

### Loss of nuclear but not cytoplasmic BAP1 protein enhanced HCC metastasis

**Fig. 3:**
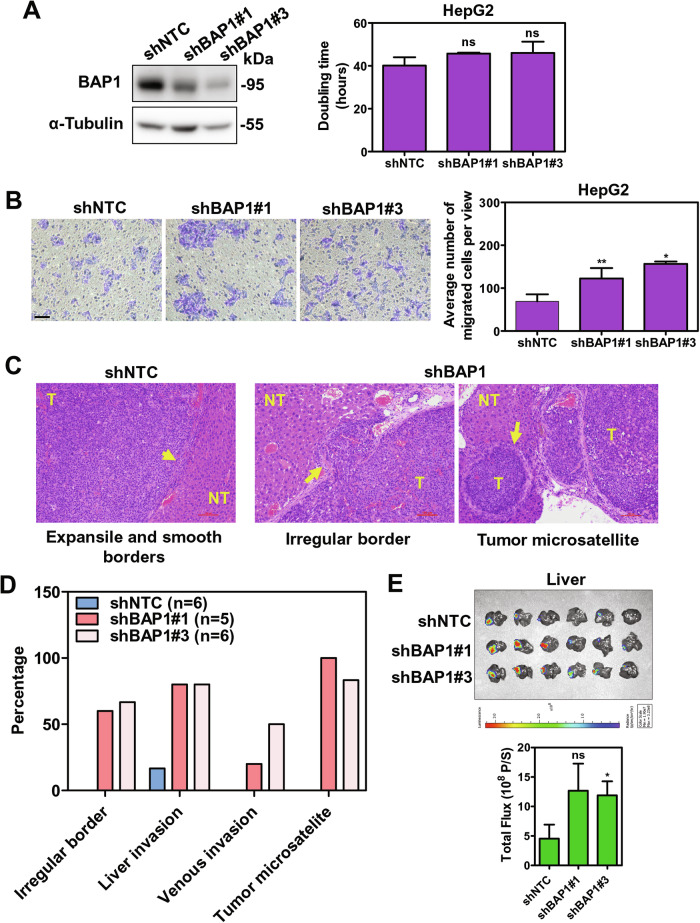
Knockdown of BAP1 promoted migratory ability but had no effect on cell proliferation in HepG2 cells. **A** Doubling time upon knockdown BAP1 protein in HepG2 cells (*n* = 3). **B** Cell migration assay upon knockdown of BAP1 in HepG2 cells. Scale bar, 100 μm (*n* = 4). **C** Representative histology of Hematoxylin & Eosin (H&E)-stained sections of livers. Short arrow indicates expansile and smooth borders of shNTC xenografts; long arrows indicate irregular tumor growth front (middle panel) and tumor microsatellite formation (right panel) in shBAP1 xenografts. Scale bar, 100 μm. **D** Frequencies of various tumor characteristic upon BAP1 knockdown detected by H&E stain. **E** Bioluminescence signal of the dissected mouse liver after orthotopic liver injection of shBAP1 HepG2 cells in nude mice (*n* = 6). (ns = *P* > 0.05, *P < 0.05, **P < 0.01, Student’s t-test).

Nuclear BAP1 expression was clearly detected in HepG2 cells (Fig. 2D, E). Interestingly, upon knockdown of BAP1 in HepG2 cells (Fig. 3A) hence resulting in loss of predominantly nuclear BAP1 protein, there was significant increase in cell migratory ability using the transwell assay, as compared with non-target control (shNTC) (Fig. 3B), while upon knockdown of BAP1 in PLC/PRF/5 and Huh7 cells, which had mainly cytoplasmic expression of BAP1, the cell migratory ability remained similar to the NTC (Fig. S1).

To investigate the functional role of nuclear BAP1 in HCC, we also employed orthotopic liver injection mouse model. We used shNTC or shBAP1 HepG2 cells tagged with luciferase to inoculate into the left lobe of liver of nude mice to examine the tumor growth and intrahepatic metastasis in mice. Of note, knockdown of BAP1 promoted intrahepatic tumor metastasis, with both BAP1 knockdown groups (shBAP1#1 and shBAP1#3) showing more frequent irregular tumor borders with invasion into the adjacent liver parenchyma, more frequent tumor microsatellite formation and venous invasion (Fig. 3C, D). There was significant increase in tumor size upon BAP1 knockdown in shBAP#3 but not with shBAP1#1, as compared with the shNTC group (Fig. 3E).

### Transcriptome sequencing identified RHOJ as a potential downstream target suppressed by BAP1 expression

**Fig. 4:**
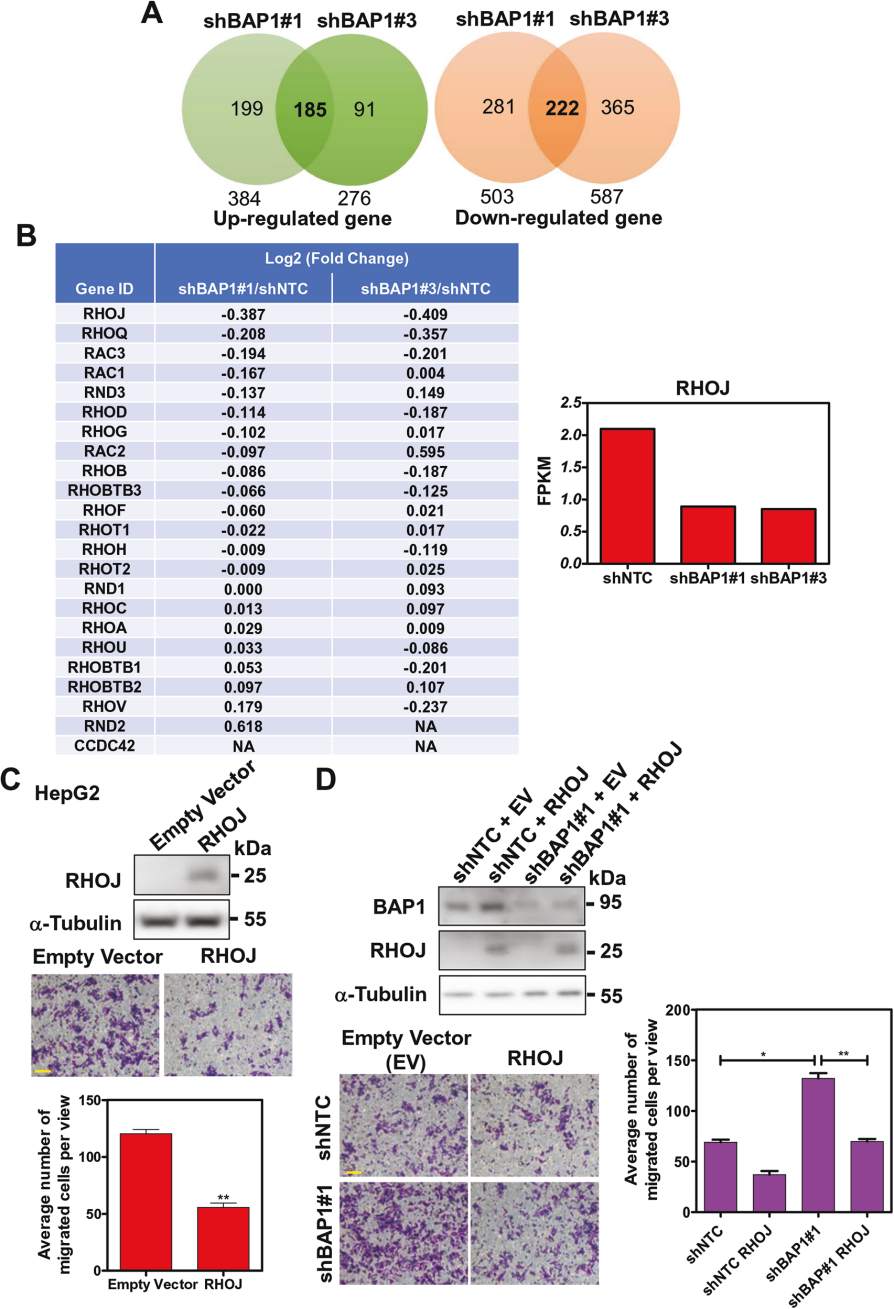
RHOJ was suppressed upon BAP1 knockdown in HepG2 cells. **A** Transcriptome analysis of BAP1 knockdown in HepG2 cells. A total of 185 genes were upregulated (left panel) and 222 genes downregulated (right panel) upon BAP1 knockdown in HepG2 cells. **B** Transcriptome expression level changes of Rho protein family upon knockdown of BAP1 in HepG2 cells (left panel). RHOJ expression level in HepG2 cells with BAP1 knockdown (right panel). **C** Cell migration assay upon overexpression of RHOJ in HepG2 cells (*n* = 3). Scale bar, 100 μm. (*P < 0.05, Student’s t-test). **D** Overexpression of RHOJ in shBAP1 HepG2 cells resulted in significantly reduced cell migratory ability as compared with the shBAP1#1 cells (*n* = 3). Scale bar, 100 μm. (*P < 0.05, **P < 0.01, Student’s t-test).

Previous reports have shown that BAP1 protein is involved in gene transcriptional regulation by removing mono-ubiquitins on histone H2AK119 [14], which is one of epigenetic modifications on chromosomes. Thus, to find the BAP1 downstream targets, we performed transcriptomic sequencing in HepG2 cells with shBAP1. A total of 185 genes had upregulated expression by 2 folds and a total of 222 genes had downregulated expression by 2 folds (Fig. 4A). Since from our afore-mentioned in vitro and in vivo experimental findings that BAP1 was more involved in HCC metastasis, we focused on the Rho protein family, which is one of the important regulators in controlling cell motility. We found that RHOJ was among the Rho protein family that were downregulated upon knockdown of BAP1 (Fig. 4B). RHOJ was previously reported to be associated with cancer metastasis [15–18]. Therefore, we investigated if RHOJ played a role in the migratory ability of HCC cells. To this end, we overexpressed RHOJ in HepG2 (Fig. 4B) cells. The overexpression of RHOJ suppressed the cell migration (Fig. 4C). The data suggest that the pro-migratory/pro-metastatic effects upon reduced BAP1 expression might be through the downregulation of RHOJ. Furthermore, upon RHOJ rescue in BAP1-knockdown HepG2 cells, the increase in cell migration induced in shBAP1#1 cells was significantly abolished, suggesting that RHOJ participates in suppressing cell mobility in HCC cells (Fig. 4D).

### OGT physically bound BAP1 and maintained BAP1 nuclear localization in HCC cells

**Fig. 5:**
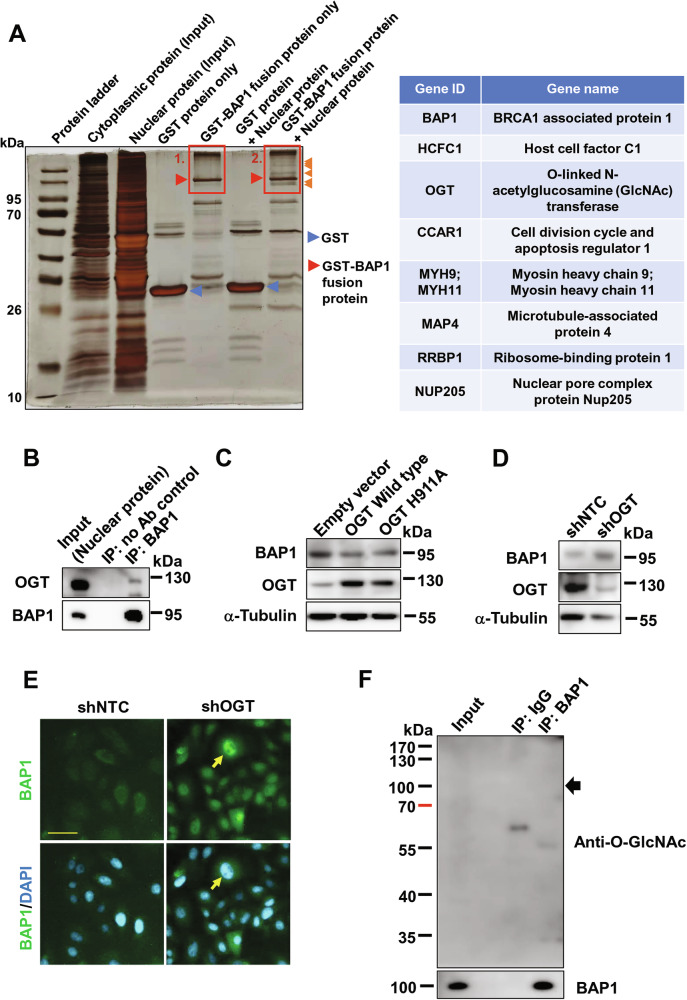
OGT physically interacted with BAP1 resulting in nuclear degradation of BAP1. **A** GST pull-down assay with silver staining using BAP1-GST fusion protein. Blue arrowheads indicate GST protein, and red arrowheads indicate BAP1-GST fusion proteins. Orange arrowheads indicate the differential bands in the group of BAP1-GST fusion protein incubated with PLC/PRF/5 nuclear protein, as compared with BAP1-GST fusion protein only group (left panel). The list of the human proteins fished out by BAP1-GST fusion protein upon incubation with PLC/PRF/5 nuclear proteins, as compared with GST protein alone (right panel). **B** Co-immunoprecipitation (co-IP) assay using anti-BAP1 antibody and nuclear protein compartment of PLC/PRF/5. **C** Overexpression of OGT reduced the BAP1 protein levels in PLC/PRF/5 cells. **D** Knockdown of OGT enhanced the BAP1 protein levels. **E** Immunofluorescence staining of BAP1 upon knockdown OGT in PLC/PRF/5 cells. Arrows indicated BAP1 localized at nucleus upon knockdown OGT. Scale bar, 50 μm. **F** O-linked-N-acetylglucosamine (O-GlcNAc) modification status of nuclear BAP1 protein in PLC/PRF/5 cells using anti-O-GlcNAc antibody detection. Arrows indicate the corresponding BAP1 protein.

To search for proteins that might directly alter the stability of nuclear BAP1, we performed GST-pull down assay followed by silver staining. We observed some differential bands above 95 kDa in the group of GST-BAP1 fusion protein incubated with PLC/PRF/5 nuclear protein, as compared with the group of GST-BAP1 fusion protein only (Fig. 5A, left panel). The gel with the differential bands (denoted with red rectangular 2) was cut and submitted for liquid chromatography-mass spectrometry (LC-MS/MS), with an aim to reveal the identities of the specific bands. Upon analysis of the mass spectrometry data, a total of 87 proteins was detected, among which 30 proteins were denoted as human proteins and 57 proteins were from *E. coli*. Of these 30 human proteins detected, 8 proteins showed enrichment upon incubation with the nuclear protein (Fig. 5A, right panel), while 22 proteins had higher levels in the GST-BAP1 fusion protein alone group, which likely represented background noise. Of note, O-linked N-acetylglucosamine (GlcNAc) transferase (OGT) was the only protein among these 8 proteins that might be related to the ubiquitin proteasome system [19]. From the experiments, we demonstrated the direct interaction between BAP1 and OGT using in vitro GST-pull down assay followed with mass spectrophotometry, and the data was in accordance with previous report that OGT binds BAP1 protein [20]. However, the influence on the stability of nuclear BAP1 is largely unknown. Hence, we further proceeded to investigate the role of OGT on the stability of nuclear BAP1.

To confirm the physical association between BAP1 and OGT in HCC, co-immunoprecipitation (co-IP) assays using BAP1 antibody and endogenous nuclear proteins from PLC/PRF/5 was performed. The results positively showed that BAP1 was physically associated with OGT in the nucleus (Fig. 5B). It has been reported that OGT has O-GlcNAc transferase activity to influence the stability of target substrate [21]. Upon overexpression of OGT and OGT H911A (an O-GlcNAc transferase-dead mutant) in PLC/PRF/5 cells, BAP1 protein level was downregulated (Fig. 5C), whereas knockdown of OGT upregulated BAP1 protein level by Western blotting and immunofluorescence (Fig. 5D, E, respectively). We further investigated whether BAP1 was modified by OGT via O-GlcNAc modification, by using a specific anti-O-GlcNAc antibody which detects O-GlcNAc modified structure in protein by Western blotting. However, upon purified BAP1 from nuclear protein lysate by immunoprecipitation and further detection by Western blotting using the anti-O-GlcNAc antibody, no observable band representing BAP1 protein could be detected (Fig. 5F), suggesting that loss of nuclear BAP1 is independent of the O-GlcNAc activity of OGT. Taken altogether, these findings suggest that the absence of nuclear BAP1 in HCC might be related to OGT.

### Both BAP1 and OGT are under NRF1 and CTCF transcriptional control in HCCs

**Fig. 6:**
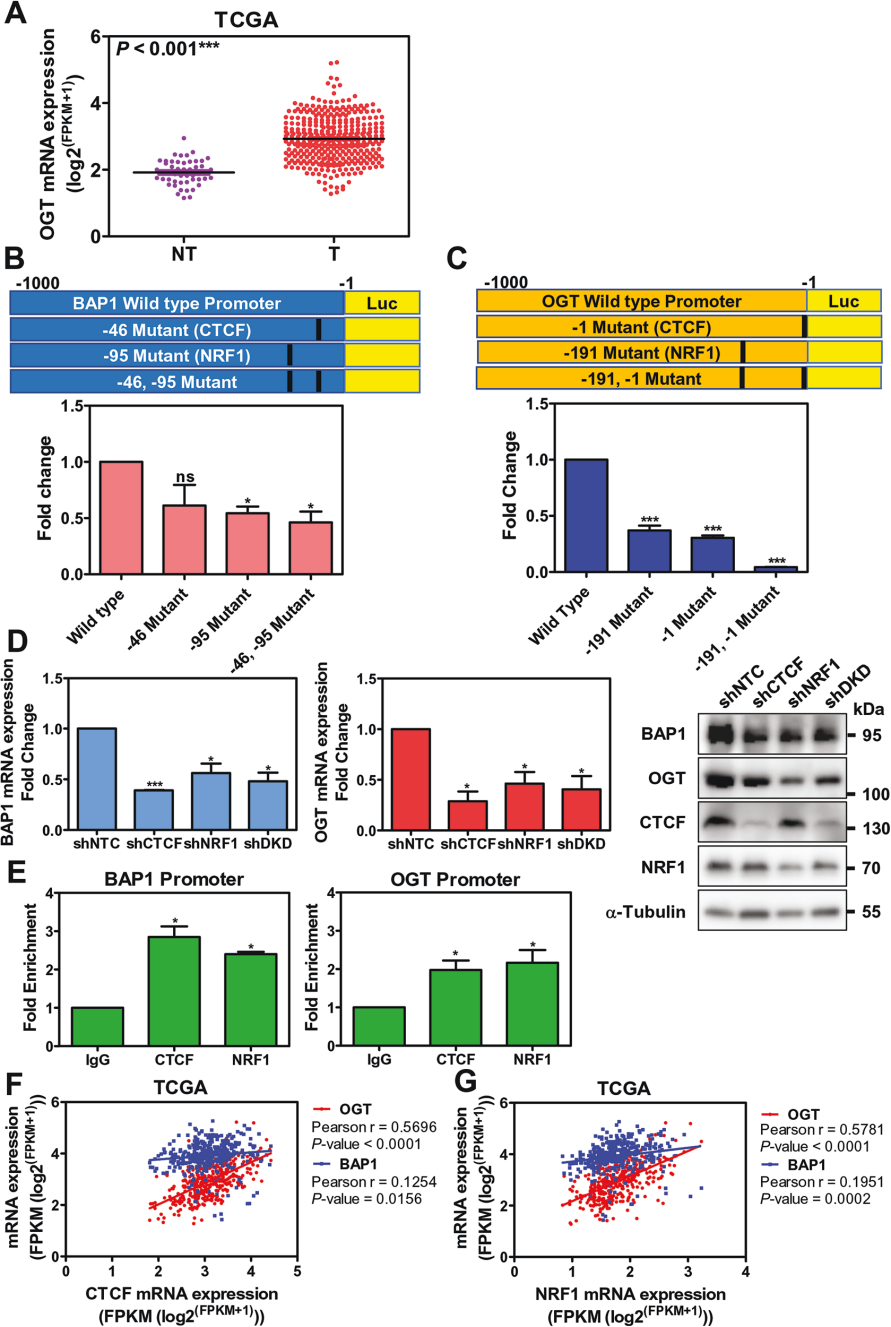
CTCF and NRF1 were transcriptional regulators of BAP1 and OGT in human HCCs. **A** OGT mRNA is upregulated in TCGA-LIHC cohort. **B** Schematic diagram of the wild-type BAP1 promoter and BAP1 promoter with mutations of the two putative transcription factor binding motifs (CTCF at −46 nt in BAP1 promoter; NRF1 at −95 nt in BAP1 promoter; and combined mutations at both regions of the BAP1 promoters) (upper panel). Dual luciferase reporter assays of BAP1 promoters of wild type and with the corresponding mutations in PLC/PRF/5 (lower panel) (*n* = 3). **C** Schematic diagram of the wild-type OGT promoter and promoters with two mutations of putative transcription factor binding motifs (CTCF at -1 nt in OGT promoter; NRF1 at -191 nt in OGT promoter; and combined mutations at both regions of the BAP1 promoters) (upper panel). Dual luciferase reporter assays of OGT promoters of wild type and with the corresponding mutations in PLC/PRF/5 (lower panel). **D** BAP1 and OGT expression upon single or double knockdown of CTCF and NRF1 in PLC/PRF/5 cells (*n* = 3). **E** ChIP assays using specific antibodies against CTCF and NRF1 in PLC/PRF/5 cells. The results confirmed both transcription factors specifically bound to and were enriched on both BAP1 and OGT promoters (*n* = 4). (ns = *P* > 0.05, *P < 0.05, **P < 0.01, *** *P* < 0.001, **** *P* < 0.0001, Student’s t-test) **F** Correlation analysis among the expression of BAP1, OGT and CTCF at mRNA level from TCGA-LIHC cohort (*n* = 371). **G** Correlation analysis among the expressions of BAP1, OGT, and NRF1 at mRNA level from TCGA HCC cohort (*n* = 371).

Although both BAP1 and OGT mRNA (Fig. 6A) were upregulated in human HCCs from TCGA-LIHC cohort, the transcriptional control of BAP1 and OGT in HCC are largely unknown. To investigate their transcriptional control, we extracted the ChIP sequencing data from the Encyclopedia of DNA Elements data portal [22] to search putative transcription factors which physically interact with BAP1 and OGT promoter region in context of liver tissue or in HepG2 cells. In addition, we further analyzed the putative consensus DNA binding site on BAP1 and OGT promoter region using the IN-silico SEarch for Co-occurring Transcription factors 2.0 [23]. From these two analyses, we identified two potential transcription factors (CTCF and NRF1) for further validation of their functional role in binding BAP1 promoter and regulating its activity. To this end, we generated (1) both wild-type BAP1 and OGT promoters, (2) mutants on putative CTCF binding site on −46 nt of BAP1 promoter and −1 nt of OGT promoter, (3) mutants on putative NRF1 binding site on −95 nt of BAP1 promoter and −191 nt of OGT promoter, and (4) double mutants on both putative CTCF and NRF1 binding sites on BAP1 and OGT promoters to conduct the luciferase reporter assays. Upon mutations of both putative CTCF and NRF1 binding sites on both BAP1 and OGT promoters, a significant reduction of the promoter activity (53.9% and 84.9% respectively) as compared with corresponding wild-type control (Fig. 6B, C). In addition, with either single knockdown of CTCF and NRF1, or double knockdown of both transcription factors by shRNA, there was an observable reduction in both BAP1 and OGT at mRNA level and protein level (Fig. 6D). Moreover, with ChIP qRT-PCR examination, there was a significant enrichment of the corresponding DNA binding motif of both CTCF and NRF1 in PLC/PRF/5 cells (Fig. 6E). Furthermore, with TCGA-LIHC cohort correlation analysis, there was significant and positive correlation between both BAP1 and OGT with CTCF (Fig. 6F) and NRF1 (Fig. 6G) at mRNA level. Collectively, the physical interaction of CTCF and NRF1 with BAP1 and OGT promoters enhanced both BAP1 and OGT transcriptional activities in HCCs.

## Discussion

In this study, we showed that BAP1 was upregulated at mRNA level in human HCC tumors from TCGA-LIHC database and our in-house RNA sequencing cohort and also with qRT-PCR analysis. The upregulation of BAP1 occurred even at early stages of HCC progression. With clinicopathological analysis, the upregulation of BAP1 mRNA expression was correlated with more aggressive tumor features and poorer patient overall survival. Intriguing, high expression of BAP1 protein was only detected in the cytoplasm but not the nucleus of tumor cells in human HCC tumors. Moreover, BAP1 protein was largely localized in the cytoplasm of HCC cell lines. Functionally, the knockdown of BAP1 in HCC cells had no significant effects on their proliferation but enhanced their migratory ability in certain types of HCC cells with mainly nuclear BAP1 protein expression such as HepG2 cells. Consistently, the knockdown of BAP1 promoted intrahepatic metastasis in HepG2 cells in vivo.

Subsequently, with RNA sequencing on stable BAP1-knockdown HepG2 cells, RHOJ was identified among the gene targets that were downregulated upon BAP1 knockdown. Overexpression of RHOJ suppressed cell migratory ability in HCC cells. RHOJ belongs to Rho-related GTP-binding protein family that regulates cell cytoskeleton remodeling and cell motility. Recent studies have suggested that RHOJ is preferentially expressed in epithelial-to-mesenchymal transition cells, with interaction with proteins (FLNB, TLN1 and IPO9) that regulate nuclear actin and inhibit actin polymerization in cancer cells [24]. Our data in this study suggests that RHOJ might be the downstream target of BAP1 in the biological activity of migration. On the other hand, the important function of nuclear BAP1 is to catalyze the removal of monoubiquitination from the histone H2A and thus regulate gene transcription [5]. However, whether BAP1 can positively regulate the transcription of RHOJ via removing the monoubiquitination of histone H2A remains to be further delineated.

**Fig. 7:**
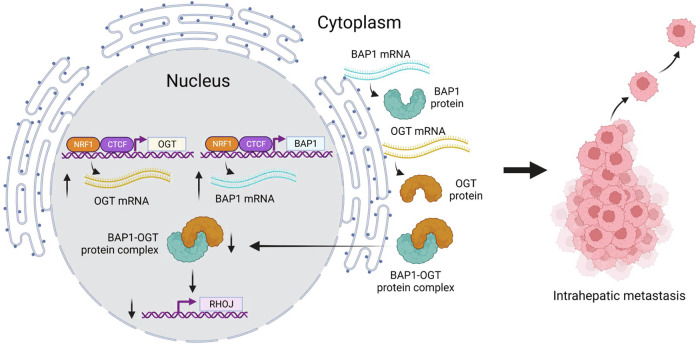
Summary of the study. Created with BioRender.com.

Regarding the regulation of BAP1 at the transcriptional level in HCC, we identified two transcription factors, CTCF and NRF1, which positively regulated BAP1 transcription by direct binding to the promoter region of BAP1. On the other hand, we found that OGT physically bound BAP1 and negatively regulated BAP1 stability in the nuclei of HCC cells in an O-GlcNAcylation independent manner. A study has reported that OGT physically interacts with the C-terminus region of BAP1, and this might mask the putative nucleus localization of BAP1, resulting in maintenance of cytoplasm subcellular localization in HCC cells [12, 20]. This might alter the interaction with other proteins, which can stabilize BAP1 [25], resulting in facilitation of BAP1 protein degradation. In other words, the reduced level of nuclear BAP1 protein in HCC might be the consequence of up-regulation of OGT in HCC. OGT is a well-known protein associated with BAP1 in the BAP1 core complex, which include ASXL1, ASXL2, HCF1, OGT, and the forkhead transcription factors FOXK1 and FOXK2 [9], and BAP1 can deubiquitinate and stabilize OGT [26]. Reversely, OGT mediates the O-GlcNAcylation of BAP1 by interacting with BAP1 C-terminal region [20]. However, we did not detect the O-GlcNAcylation of BAP1 in HCC cells. Recently, it has been reported that O-GlcNAcylation plays a role in enhancing HCC formation in mice via a high dietary fructose intake; this implies that OGT is important in promoting cancer cell survival. However, how OGT governs HCC progression remains to be further investigated [27]. Surprisingly, we found that the BAP1 and OGT transcription was both upregulated and under control of CTCF and NRF1 in vitro and in human HCC, suggesting that both positive and negative factors, which influence BAP1 nuclear localization in HCC, are present. In the present study, we observed that both BAP1 and OGT were overexpressed in HCC tissues. BAP1 has been reported to serve as an epigenetic regulator in cancer [28]. OGT might serve as a negative regulator, via controlling BAP1 stability, to indirectly control the epigenetic reprogramming in HCC. To summarize, this study uncovered the underlying mechanisms of how BAP1 was upregulated at mRNA. Loss of the nuclear localization of BAP1 protein contributed to enhanced cell migration and promoted more aggressive tumor behavior in human HCCs (Fig. 7).

## Supplementary information

Supplementary Materials

Original Western blot

## Data Availability

The results present here are in whole or part based upon data generated by the TCGA Research Network (https://www.cancer.gov/tcga) or extracted from UCSC Xena browser (https://xena.ucsc.edu/). Other data needed to evaluate the conclusions in the paper are present in the paper and/or the Supplementary Materials. All materials are available upon request to the corresponding authors.

## References

[1] Llovet JM, Zucman-Rossi J, Pikarsky E, Sangro B, Schwartz M, Sherman M, et al. Hepatocellular carcinoma. Nat Rev Dis Primers. 2016;2:16018. 27158749 10.1038/nrdp.2016.18

[2] Villanueva A. Hepatocellular Carcinoma. N Engl J Med. 2019;380:1450–62. 30970190 10.1056/NEJMra1713263

[3] Asafo-Agyei KO, Samant H. Hepatocellular Carcinoma. StatPearls [Internet]. Treasure Island (FL): StatPearls Publishing; 2025. 32644603

[4] Jensen DE, Proctor M, Marquis ST, Gardner HP, Ha SI, Chodosh LA, et al. BAP1: a novel ubiquitin hydrolase which binds to the BRCA1 RING finger and enhances BRCA1-mediated cell growth suppression. Oncogene. 1998;16:1097–112. 9528852 10.1038/sj.onc.1201861

[5] Carbone M, Yang H, Pass HI, Krausz T, Testa JR, Gaudino G. BAP1 and cancer. Nat Rev Cancer. 2013;13:153–9. 23550303 10.1038/nrc3459 PMC3792854

[6] Brugarolas J. Molecular genetics of clear-cell renal cell carcinoma. J Clin Oncol. 2014;32:1968–76. 24821879 10.1200/JCO.2012.45.2003 PMC4050206

[7] Pena-Llopis S, Vega-Rubin-de-Celis S, Liao A, Leng N, Pavia-Jimenez A, Wang S, et al. BAP1 loss defines a new class of renal cell carcinoma. Nat Genet. 2012;44:751–9. 22683710 10.1038/ng.2323 PMC3788680

[8] Machida YJ, Machida Y, Vashisht AA, Wohlschlegel JA, Dutta A. The deubiquitinating enzyme BAP1 regulates cell growth via interaction with HCF-1. J Biol Chem. 2009;284:34179–88. 19815555 10.1074/jbc.M109.046755 PMC2797188

[9] Yu H, Mashtalir N, Daou S, Hammond-Martel I, Ross J, Sui G, et al. The ubiquitin carboxyl hydrolase BAP1 forms a ternary complex with YY1 and HCF-1 and is a critical regulator of gene expression. Mol Cell Biol. 2010;30:5071–85. 20805357 10.1128/MCB.00396-10 PMC2953049

[10] Misaghi S, Ottosen S, Izrael-Tomasevic A, Arnott D, Lamkanfi M, Lee J, et al. Association of C-terminal ubiquitin hydrolase BRCA1-associated protein 1 with cell cycle regulator host cell factor 1. Mol Cell Biol. 2009;29:2181–92. 19188440 10.1128/MCB.01517-08 PMC2663315

[11] Ji Z, Mohammed H, Webber A, Ridsdale J, Han N, Carroll JS, et al. The forkhead transcription factor FOXK2 acts as a chromatin targeting factor for the BAP1-containing histone deubiquitinase complex. Nucleic Acids Res. 2014;42:6232–42. 24748658 10.1093/nar/gku274 PMC4041447

[12] Ventii KH, Devi NS, Friedrich KL, Chernova TA, Tighiouart M, Van Meir EG, et al. BRCA1-associated protein-1 is a tumor suppressor that requires deubiquitinating activity and nuclear localization. Cancer Res. 2008;68:6953–62. 18757409 10.1158/0008-5472.CAN-08-0365 PMC2736608

[13] Wang F, Luo M, Qu H, Cheng Y. BAP1 promotes viability and migration of ECA109 cells through KLF5/CyclinD1/FGF-BP1. FEBS Open Bio. 2021;11:1497–503. 33529461 10.1002/2211-5463.13105 PMC8091813

[14] Carbone M, Harbour JW, Brugarolas J, Bononi A, Pagano I, Dey A, et al. Biological mechanisms and clinical significance of BAP1 mutations in human cancer. Cancer Discov. 2020;10:1103–20. 32690542 10.1158/2159-8290.CD-19-1220 PMC8006752

[15] Zhang Z, Chen B, Zhu Y, Zhang T, Zhang X, Yuan Y, et al. The Jumonji Domain-Containing Histone Demethylase Homolog 1D/lysine Demethylase 7A (JHDM1D/KDM7A) is an epigenetic activator of RHOJ transcription in breast cancer cells. Front Cell Dev Biol. 2021;9:664375. 34249916 10.3389/fcell.2021.664375 PMC8262595

[16] Nozaki M, Nishizuka M. Repression of RhoJ expression promotes TGF-beta-mediated EMT in human non-small-cell lung cancer A549cells. Biochem Biophys Res Commun. 2021;566:94–100. 34119829 10.1016/j.bbrc.2021.06.004

[17] Ho H, Soto Hopkin A, Kapadia R, Vasudeva P, Schilling J, Ganesan AK. RhoJ modulates melanoma invasion by altering actin cytoskeletal dynamics. Pigment Cell Melanoma Res. 2013;26:218–25. 23253891 10.1111/pcmr.12058 PMC4528913

[18] Kim C, Yang H, Park I, Chon HJ, Kim JH, Kwon WS, et al. Rho GTPase RhoJ is Associated with Gastric cancer progression and metastasis. J Cancer. 2016;7:1550–6. 27471571 10.7150/jca.15578 PMC4964139

[19] Ruan HB, Nie Y, Yang X. Regulation of protein degradation by O-GlcNAcylation: crosstalk with ubiquitination. Mol Cell Proteom. 2013;12:3489–97. 10.1074/mcp.R113.029751 PMC3861702 23824911

[20] Moon S, Lee YK, Lee SW, Um SJ. Suppressive role of OGT-mediated O-GlcNAcylation of BAP1 in retinoic acid signaling. Biochem Biophys Res Commun. 2017;492:89–95. 28802580 10.1016/j.bbrc.2017.08.029

[21] He X, Li Y, Chen Q, Zheng L, Lou J, Lin C, et al. O-GlcNAcylation and stablization of SIRT7 promote pancreatic cancer progression by blocking the SIRT7-REGgamma interaction. Cell Death Differ. 2022;29:1970–81. 35422493 10.1038/s41418-022-00984-3 PMC9525610

[22] Luo Y, Hitz BC, Gabdank I, Hilton JA, Kagda MS, Lam B, et al. New developments on the Encyclopedia of DNA Elements (ENCODE) data portal. Nucleic Acids Res. 2020;48:D882–D9. 31713622 10.1093/nar/gkz1062 PMC7061942

[23] Rohr CO, Parra RG, Yankilevich P, Perez-Castro C. INSECT: IN-silico SEarch for Co-occurring Transcription factors. Bioinformatics. 2013;29:2852–8. 24008418 10.1093/bioinformatics/btt506

[24] Debaugnies M, Rodriguez-Acebes S, Blondeau J, Parent MA, Zocco M, Song Y, et al. RHOJ controls EMT-associated resistance to chemotherapy. Nature. 2023;616:168–75. 36949199 10.1038/s41586-023-05838-7 PMC10076223

[25] Wang L, Birch NW, Zhao Z, Nestler CM, Kazmer A, Shilati A, et al. Epigenetic targeted therapy of stabilized BAP1 in ASXL1 gain-of-function mutated leukemia. Nat Cancer. 2021;2:515–26. 35122023 10.1038/s43018-021-00199-4

[26] Dey A, Seshasayee D, Noubade R, French DM, Liu J, Chaurushiya MS, et al. Loss of the tumor suppressor BAP1 causes myeloid transformation. Science. 2012;337:1541–6. 22878500 10.1126/science.1221711 PMC5201002

[27] Zhou P, Chang WY, Gong DA, Xia J, Chen W, Huang LY, et al. High dietary fructose promotes hepatocellular carcinoma progression by enhancing O-GlcNAcylation via microbiota-derived acetate. Cell Metab. 2023;35:1961–75 e6. 37797623 10.1016/j.cmet.2023.09.009

[28] Caporali S, Butera A, Amelio I. BAP1 in cancer: epigenetic stability and genome integrity. Discov Oncol. 2022;13:117. 36318367 10.1007/s12672-022-00579-x PMC9626716

