# Supplementary material for: Nuclear localization of BRCA1-associated protein 1 is important in suppressing hepatocellular carcinoma metastasis via CTCF and NRF1/OGT axis
Source: Cell Death Dis. 2025 Feb 21;16(1):123. doi: 10.1038/s41419-025-07451-0 (PMC11845619; doi:10.1038/s41419-025-07451-0)
Supplement: Supplementary file 1 — Supplementary Materials [file 41419_2025_7451_MOESM1_ESM.docx]

**Supplementary information**

**
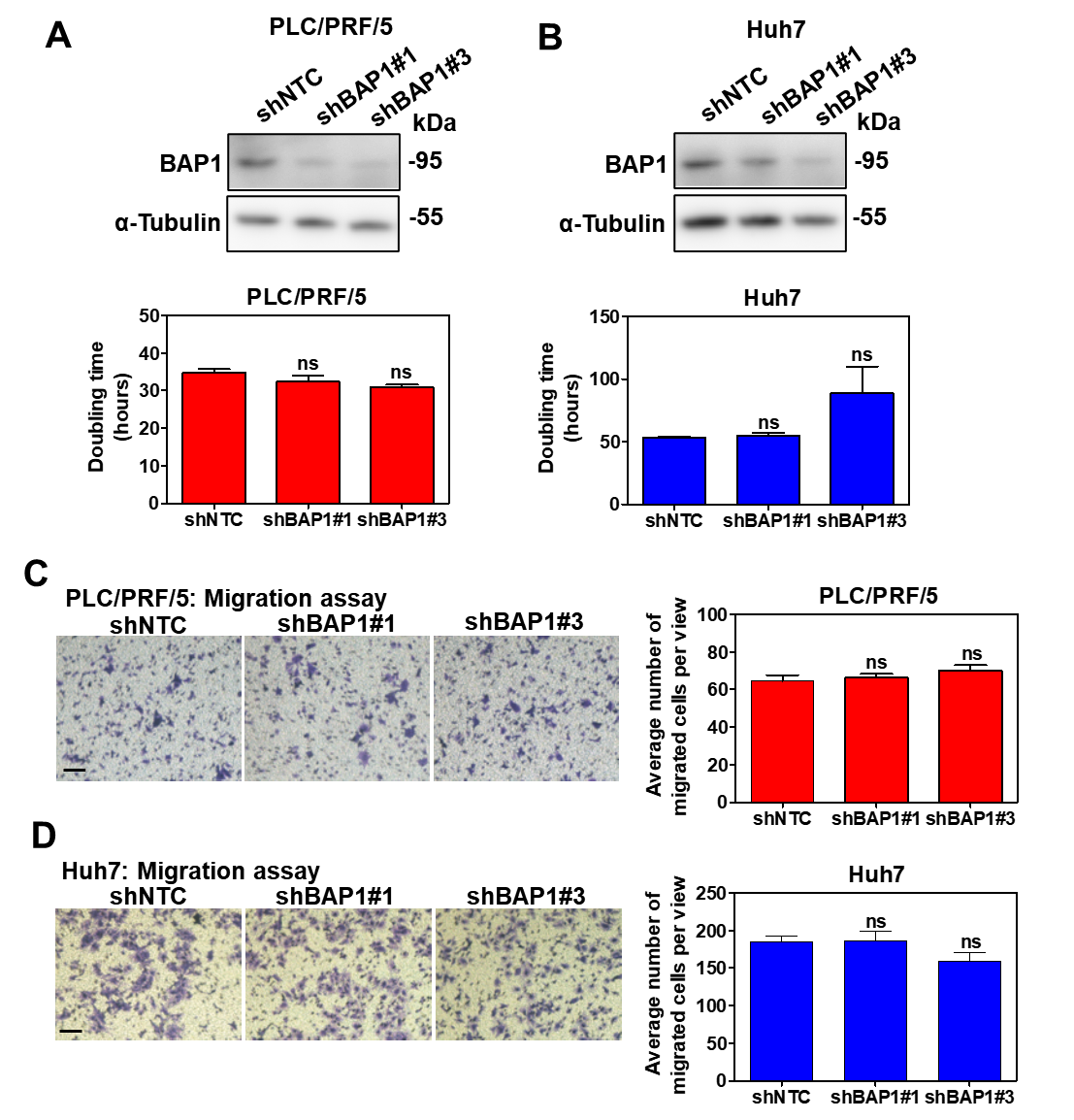
**

**Supplementary Fig S1.** Knockdown of BAP1 did not promote migratory ability and cell proliferation in both PLC/PRF/5 and Huh7 cells. A and B. Doubling time upon knockdown BAP1 protein in PLC/PRF/5 and Huh7 cells (n=3). C and D. Cell migration assay upon knockdown of BAP1 in PLC/PRF/5 and Huh7 cells. Scale bar, 100 μm (n=3), (ns=*P*>0.05, Student’s t-test)

**
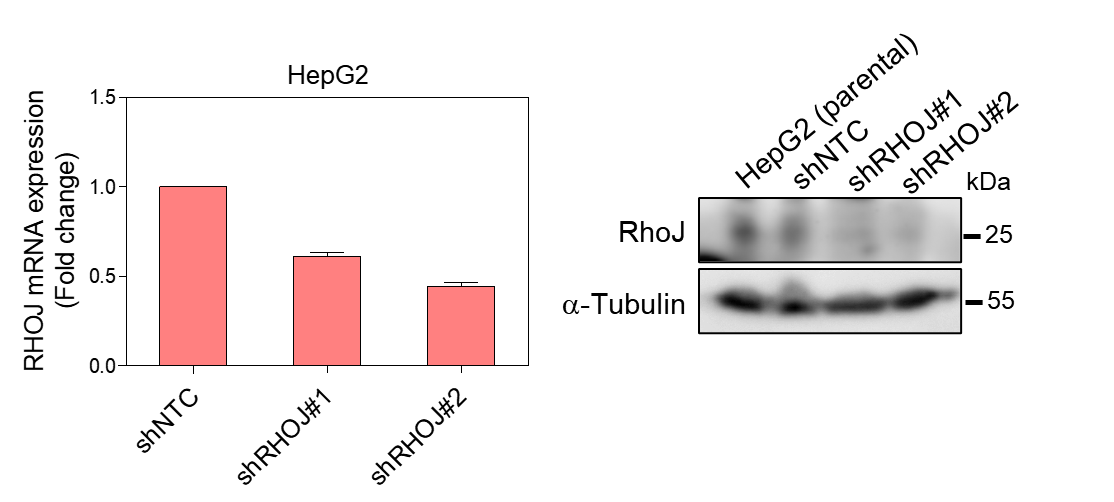
**

**Supplementary Fig S2.** Western blotting showing the endogenous RHOJ expression in HepG2 cells together with shRHOJ in HepG2 cells, using the same antibody for detection in this study as reference.

**Materials and Methods**

**Patient Samples**

HCC samples from 124 patients who underwent surgical resection of tumors at Queen Mary Hospital (QMH), Hong Kong were randomly selected. All specimens were obtained immediately after surgical resection, snap frozen in liquid nitrogen, and kept at -80°C or formaldehyde fixed and parafilm embedded as tumor blocks, stained for histological examination and immunohistochemistry. This study was approved by the institutional review board of the University of Hong Kong/Hospital Authority Hong Kong West Cluster (UW 17-056) and informed consents from patients.

**Cell lines**

Human HCC cells, HepG2, Hep3B, PLC/PRF/5 and SNU449 were obtained from American Type Culture Collection. Huh7 and HLE were purchased from the Japanese Collection of Research Bioresources Cell Bank. Human HCC cell line MHCC97L was a gift from Dr. ZY Tang (Fudan University, Shanghai, China) and the STR authentication was conducted (Fig S3). Immortal liver cell line MIHA was obtained from Dr. J Roy-Chowdhury (Albert Einstein College of Medicine, New York, USA). Human embryonic kidney cell line 293FT was obtained from Invitrogen (Carlsbad, USA). HepG2 and PLC/PRF/5 were cultured in Minimum Essential Medium containing 10% fetal bovine serum and 1mM sodium pyruvate, SNU449 was cultured in RPMI-1640 Medium containing 10% fetal bovine serum, while the others were cultured in Dulbecco’s modified Eagle’s medium high glucose supplemented with 10% fetal bovine serum.

**Plasmids**

Full-length BAP1 open reading frame which subcloned into pLVX-M-Puro vector (Addgene plasmid # 125840; http://n2t.net/addgene:125840; RRID:Addgene 125840) and pLVX-M-Puro vector (Addgene plasmid # 125839; http://n2t.net/addgene:125839; RRID:Addgene_125839) were gift from Boyi Gan. Full-length forms of CTCF, NRF1 and RHOJ ORF were amplified from HepG2 complementary DNA (cDNA) and subcloned into vector Flag/pcDNA3.1+neo. Full-length form of OGT was amplified from HepG2 cDNA and subcloned into vector Myc/pLVX-M_Puro. Moreover, the BAP1 promoter (chr 3. 52,411,030-52,410,031) and OGT promoter (chr X. 71,532,104-71,533,103) were amplified from HepG2 genomic DNA and subcloned into pGL3-Basic vector. A series of BAP1 or OGT promoter DNA fragments with mutations were amplified from wild-type BAP1 or OGT promoter/ pGL3-Basic vector and sub-cloned into the empty vector of pGL3-Basic. In addition, short-hairpin (sh) RNA interfering sequences targeting CTCF, NRF1, OGT and BAP1, were purchased from Horizon Discovery (Cambridge, United Kingdom) or Sigma-Aldrich (Burlington, MA) were synthesized by Integrated DNA Technologies (Coralville, IA) and subcloned into the pLKO.1-Puro vector. The primers used for cloning are listed in Supplementary Table S1.

**Tissue microarrays and immunohistochemistry**

The paraffin blocks of the tissue microarrays comprised of many paraffin cores each of which is about 1-2 mm in diameter from different cases and at defined array coordinates. The technique allows analysis of multiple samples in a single slide and avoids discrepancy caused by manipulation to a large extent. For each case, there were 2 cores for each of the HCC tumor tissue and the corresponding non-tumorous liver tissue. Immunohistochemistry of BAP1 was conducted by using anti-BAP1 antibody (sc-28383, Santa Cruz biotechnology, 1:100) with standard protocol. The slides were scanned and analyzed by Nanozoomer digital scan system (Hamamatsu Photonics K.K, Japan) according to the manufacturer’s instructions.

**Dual luciferase reporter assay**

HepG2 and Huh7 cells were transfected with different combinations of plasmids using Lipofectamine 3000 (Invitrogen) according to the manufacturer’s protocol. The plasmids included BAP1 wild type promoter/pGL3-Basic, OGT wild type promoter/pGL3-Basic, various forms of mutated BAP1 and OGT promoter/pGL3-Basic reporter constructs, and an internal control pRL/PGK. Twenty-four hours after transfection, luciferase and Renilla luciferase activities were measured by a Dual Luciferase Reporter assay system (Promega, Madison, WI) according to the manufacturer’s protocol. Transfection efficiency was normalized with the Renilla luciferase activity. Experiments were done at least three times independently.

**Western blot analysis**

Cells were lyzed in sodium dodecyl sulfate (SDS)-containing buffer and equal amounts of protein were separated in SDS-polyacrylamide gel electrophoresis gel for western blot analysis. Immunodetection was performed using antibodies that were listed in Supplementary Table S2.

**RNA extraction, cDNA synthesis and qPCR analysis**

Total RNA was extracted from the paired HCCs and their corresponding non-tumor liver tissue (NTL) tissues using Trizol reagent (Invitrogen). The cDNA synthesis using GeneAmp PCR Reagent Kit (Applied Biosystems, Foster City, CA) was used to reverse messenger RNA into cDNA. The FAM TaqMan probe Hs01109276_g1 (Applied Biosystems) was used to detect the BAP1 mRNA expression in human tissue, and the VIC TaqMan probe Hs99999909_m1 (Applied Biosystems) was used to detect HPRT1 and was served as the endogenous control in the qRT-PCR analysis using ABI TaqMan Gene Expression Assay Kit (Applied Biosystems). To detect OGT mRNA in cell lines, the primer set with forward primer 5’- AGCACAGAACCAACGAAACG-3’ and reverse primer 5’- CAGCCTTCGACACTGGAAGT-3’ couple with ABI Power SYBR™ Green PCR Master Mix (Applied Biosystems) for measurement.

**Chromatin immunoprecipitation assay**

The detailed protocol has been described ^15^. The antibody against CTCF protein (ab128873, Abcam), NRF1 protein (ab175932, Abcam) and normal Mouse IgG antibody (sc-3877, Santa Cruz biotechnology) were used for the chromatin immunoprecipitation (ChIP) assay. The TaqMan probe with forward primer (5’- CTCGCCCCACCTGCGCCCAG-3’), reverse primer (5’- ACCAAGGCGCGACGGCCCTG-3’) and FAM-conjugated probe (5’- CGCGTGCGCGTTCGCCTTCGA-3’) covering the putative CTCF and NRF1 binding site on BAP1 and the TaqMan probe with forward primer (5’- CAGAGAGTGCCCTAACCACG-3’), reverse primer (5’- GGCGGCGACGGCTCCTCGGCCG-3’) and FAM-conjugated probe (5’- GGTGT TTGTCGCAATGGGC-3’) covering the putative CTCF and NRF1 binding site on OGT DNA region were employed for standard qRT-PCR using ABI TaqMan Gene Expression Assay supplements of AmpliTaq Gold 360 master mix (Applied Biosystems) and detected by ABI QuantStudio 5 Real-Time PCR System (Applied Biosystems) in the ChIP assay.

**Stable knockdown HCC cell establishment**

A lentiviral-mediated approach was used to construct stable BAP1, OGT, CTCF or NRF1 knockdown HCC cell lines. Human ON‐TARGETplus SMARTpool siRNA duplexes which targeting BAP1, CTCF and NRF1 and non-target control were purchased from Horizon Discovery while OGT obtained from Sigma-Aldrich. The pLKO.1-Puro or pLKO.1-Hygro (a gift from Bob Weinberg (Addgene plasmid # 24150; http://n2t.net/addgene:24150; RRID:Addgene 24150)) vectors contained shRNAs targeting corresponding gene targets and a non-target control were stably transduced into HCC cell lines. Puromycin or Hygromycin selection was performed to get the stable expression of shRNAs and shNTCs. Sequences of all shRNAs are listed in Supplementary Table S1.

**Stable overexpressing open reading frame HCC cell establishment**

A lentiviral-mediated approach was used to construct stable BAP1, OGT, and RHOJ overexpressing HCC cell lines. The BAP1/pLVX-M-Puro, pLVX-M-Puro vector contained Myc-tagged OGT or Flag-tagged RHOJ open reading frame and empty pLVX-M-Puro control vector were stably transduced into HCC cell lines. Puromycin selection was performed to get the stable expression of corresponding protein products.

**Immunofluorescence staining**

We performed the immunofluorescence staining in a 96-well plate format. First, 5,000 cells were seeded in 96-well plate were incubated at 37°C overnight and then fixed with 3.7% formaldehyde in PBS for 30 minutes. After fixation, cells were washed once by PBS. After treatment with 0.2% Triton X-100 in PBS for 3 minutes for the purpose of permeabilization, the cells were washed with PBS. Then, 3% BSA in PBS was used for blocking. Following this, cells were incubated with freshly prepared primary antibody in 3% BSA/PBS for an hour at room temperature. Afterwards, cells were washed by PBS and incubated with fluorescein isothiocyanate (FITC) conjugated second antibody (1: 200) and 4′,6-diamidino-2-phenylindole (DAPI) (1: 1,000) for 1 hour at room temperature in dark environment. Cells were then washed 3 times by PBS at room temperature. Lastly, cells were kept in PBS and observed under inverted fluorescence microscope. Photos of immunofluorescence were taken by either Nikon Eclipse TS100-F Trinocular Inverted Fluorescence Microscope (Kabushiki-gaisha Nikon, Japan) or ImageXpress Pico Automated Cell Imaging System (Molecular Devices, San Jose, CA).

**Transwell assay**

100,000 cells were resuspended into 100 μL of serum-free medium and added to the upper chamber of the insert with 8 μm-pore size membrane (Merck Millipore, Burlington, MA). For the bottom chamber, 500 μL of standard culture medium supplemented with 40 ng/mL EGF was added to the bottom chamber of cell culture insert in the well of 24-well plate acting as chemoattractant. After incubation, cells on the insert membrane were fixed by 100% methanol for 15 minutes and stained with crystal violet solution for 20 minutes. After washing the insert with water, non-migrated cells on the upper side of the membrane were gently removed by cotton swab. Photos were taken from three randomly selected fields with migrated cells on the lower side of the membrane and cells counted manually. Experiments were done at least three independently.

**Cell proliferation assay**

Two thousand cells were seeded on each well of 96-well plate. Cells were then cultured for 5 days. For every 24 hours (Day 1 to Day 5), assigned cells were fixed by 30 μL of 100% methanol per well for 5 minutes and replaced with 100 μL/well of PBS after cell fixation to preserve cells for a short time. After 5 days, PBS was removed and diluted DAPI at 1 μg/mL was used to stain the cell nuclei. After staining, the plate was subjected to fluorescent photo taking and cell counting by the ImageXpress Pico Automated cell Imaging System (Molecular Devices). The cell counting program setting was pre-configured by manufacturer. Experiments were done at least three independently.

**Transcriptome sequencing.**

Transcriptome sequencing (RNA-seq) of shNTC and shBAP1 in HepG2 cells was performed using TruSeq standard mRNA sample Prep kit (Illumina, CA, USA) for PolyA+ mRNA library preparation. This was followed by a 2 x 150 base pair read length with 50 million paired-end reads sequencing in NovaSeq 6000 (Illumina, CA, USA) by Centre for PanorOmic Science, The University of Hong Kong. Fragments Per Kilobase Million (FPKM) output was obtained via Top Hat-Cufflinks pipeline.

**Orthotopic mouse injection model**

2×10^6^ cells were suspended in 50% Matrigel in serum-free medium and injected into the left lobe of the BALB/cAnN-nu male mouse liver. At the end point, mice were injected intraperitoneally with 100 mg/kg D-luciferin (Perkin Elmer, Waltham, MA) and imaged by bioluminescence using the IVIS Spectrum Imaging System (Perkin Elmer). Then, mice were subjected to dissection, and livers and lungs were collected and imaged. The detected intensity of bioluminescence signals reflected the burden of the mouse tumor xenografts and the extent of distant metastasis to the lung. The resected tissue was formaldehyde fixed and parafilm embedded as tissue block for microtone sectioning and Hematoxylin and Eosin staining for microscopic examination. All experimental procedures on mice were approved by the Committee on the Use of Live Animals in Teaching and Research of the University of Hong Kong (CULATR 5089-19 and 5688-21) and conducted in accordance with the Animals (Control of Experiments) Ordinance of Hong Kong.

**GST-pull down assay**

To produce BAP1-GST fusion protein, the BAP1 open reading frame was digested from BAP1/pLVX-M-Puro and subcloned to the expression vector pGEX-4T1 vector. Next, the recombinant plasmid was transformed to *Escherichia coli* cells (BL 21 strain) with Isopropyl β-D d-1-thiogalactopyranoside as an inducer. Sonication and detergent in the lysis buffer were used to break down *E. coli* cells and extraction of the fusion protein. Binding of GST fusion protein to GST beads (Glutathione Sepharose^TM^ 4 fast flow) (Cytiva, Marlborough, MA) was achieved by incubation at room temperature for 90 minutes. Afterwards, the bound GST beads were washed by PBS 5 times and preserved at 4 °C for later use by re-suspending the beads with the storage buffer (20 mM HEPES-KOH at pH 7.9, 50 mM NaCl, 1 mM MgCl_2_, 17% glycerol and 2 mM DTT). Bio-Rad Protein Assay Kit (Bio-Rad Laboratories, Hercules CA) was used to determine the protein concentration of the suspension of GST beads binding of fusion proteins. For GST pull-down assay involving mammalian cell lysate, 200 μg of protein lysate extracted from mammalian cells and 5 μg GST fusion protein with GST beads were incubated at 4°C for 3 hours with gentle rotation. Then, the protein-bound GST beads were washed 5 times with the mammalian cell protein extraction buffer. After this, the beads were resuspended by 30 μL 2× SDS-containing sample buffer and subjected to 95°C in dry bath for 5 minutes. Then, the beads were removed by centrifugation at 5,000 × g for 1 minute and the supernatant was ready for protein electrophoresis.

**Silver staining**

Silver staining of protein gel was done to show target proteins without antibody. After SDS-PAGE, the gel was rinsed twice by double distilled water (ddH_2_O) in a new 15 cm plastic dish. Then, the gel was fixed by fixation buffer (40% Ethanol, 10% Acetic acid) for 30 minutes with shaking, followed by quick rinse with ddH_2_O twice. Afterwards, the gel was washed by ddH_2_O with shaking for another 30 minutes. The similar procedure was performed for sensitizing with sensitize buffer (40% Ethanol, 500 mM NaCH₃COO, and 20 mM NA_2_S_2_O_3_). A new dish was changed for the staining process. A dark environment was maintained for subsequent steps of silver staining and development. The gel was stained with 7.36 mM AgNO_3_ for 30 minutes, followed by quick rinse with ddH_2_O twice. The developing time by developing buffer (236 mM Na_2_CO_3_ with 0.02% formaldehyde) varied for individual experiments. Finally, the reaction was terminated by 50 mM EDTA solution. To ensure the quality and performance of each experiment, ddH_2_O and solutions for staining and development were freshly prepared.

**Liquid chromatography-mass spectrometry and data analysis**

Protein samples were resolved using SDS-PAGE gel and visualized with silver stain. Both gel lanes were separately cut into slices and subjected to in-gel digestion. Briefly, gel slices were subjected to reduction and alkylation by 10 mM TCEP and 55 mM 2-chloroacetamide, respectively. Protein digestion was performed incubating with trypsin (1 ng/µl) overnight at 37 °C. Subsequent tryptic peptides were extracted from the gel with 50% ACN/5% FA and 100% ACN sequentially. The peptide extracts were pooled together and speedvac dried. The Peptides were desalted using C18 StageTips for liquid chromatography-mass spectrometry (LC-MS/MS) analysis. For data acquisition, eluted peptides were analyzed with nanoelute UHPLC was coupled to Bruker timsTOF pro mass spectrometer. The peptide mixture was loaded onto an Aurora C18 UHPLC column (75 μm i.d. × 25 cm length × 1.6 μm particle size (IonOpticks, Australia). Chromatographic separation was carried out using a linear gradient of 2-30% of buffer B (0.1% FA in ACN) at a flow rate of 300 nL/min over 27 minutes. MS data was collected over a m/z range of 100 to 1,700, and MS/MS range of 100 to 1,700. During MS/MS data collection, each TIMS cycle was 1.1 s and included 1 MS + an average of 10 PASEF MS/MS scans. Regarding the data analysis, Raw mass spectrometry data were processed using MaxQuant 1.6.14.0. Raw data was searched against *E. Coli* UniProt FASTA database (Nov 2020) containing 7, 153 entries/Human UniProt FASTA database (Apr 2020) containing 74, 824 entries, using settings as below: oxidized methionine (M), acetylation (Protein N-term) were selected as dynamic modifications, and carbamidomethyl (C) as fixed modifications with minimum peptide length of 7 amino acids was enabled. Confident proteins were identified using a target-decoy approach with a reversed database, strict false-discovery rate 1% at peptide and peptide spectrum matches (PSMs) level; minimum ≥1 unique peptide, ≥2 PSMs. Sample processing, liquid chromatography-mass spectrometry (LC-MS/MS) analysis were performed at the Proteomics and Metabolomics Core Facility, Centre for PanorOmic Sciences, the University of Hong Kong.

**Cell fractionation and immunoprecipitation**

In brief, cell lysate was acquired by incubation of the cells with the cytosolic lysis buffer (10 mM HEPES (pH 7.9), 10 mM KCL, 1 mM EDTA, 1 mM DTT and 1x complete EDTA-free protease inhibitor cocktail (Roche)) at 4°C for 30 minutes, followed by centrifugation at 16,000 x g for 5 minutes at 4°C. After removal of the supernatant, the pellet was resuspended into nuclear extraction buffer (150 mM NaCl, 5 mM EDTA, 50 mM Tris-HCl with pH 8.0, 0.1% Triton X-100 and 1 x complete EDTA-free protease inhibitor cocktail (Roche, Basel, Switzerland)) at 4°C for 30 minutes, followed by centrifugation at 16,000 x g for 5 minutes at 4°C. The supernatant was kept and the cell debris at the bottom was discarded. To precipitate target protein complex in cell lysate, 1 µg of antibody was used to incubate with 500 µg of protein in lysis buffer in 4°C cold room with gentle rotation. 30 µL of Protein G Sepharose (Cytiva) was added to the mixture of antibody and cell lysate on the next day and incubated in 4°C cold room for another 4 hours with rotation. Protein G Sepharose was pre-washed by PBS 3 times and preserved at 4°C in PBS at ration of 1: 1 (v/v). Afterwards, the Sepharose beads were washed 5 times with lysis buffer by centrifugation at 5,000 rpm for 1 minutes at 4°C and vortex. Then, the Sepharose was incubated in 95°C in dry bath for 5 minutes with 30 μL 2× SDS sample buffer. Recognition of proteins in the supernatant was achieved by Western Blot.

**Clinicopathological analysis**

Clinicopathological analysis was performed to investigate the clinicopathological significance of BAP1 expression. The clinicopathological parameters consisted of gender, venous invasion, tumor encapsulation, tumor microsatellite formation, direct liver invasion, cellular differentiation, tumor size, HBsAg status, background of liver disease, and pTNM stage. The analysis was conducted using IBM SPSS Statistics 27 software (International Business Machines Corporation, Armonk, NY). If the number of cases used for analysis in total is over 100, Chi square test was used for significance analysis, otherwise Fisher’s exact test was applied. *P*-value less than 0.05 were classified as significant.

**Statistical analysis**

Statistical analyses were performed using GraphPad Prism 5.0 software (GraphPad Software Inc., Boston, MA). One-way ANOVA with Dunnett comparison test for more than two groups or Student’s t-test was used to compare the mean values of two groups. For *in vitro* and *in vivo* experiments, data were expressed as mean ± SD followed by either Unpaired Student’s t-test or paired Student’s t-test. Log rank test for survival analysis. Statistical significance was defined as ns=*P*>0.05, **P*<0.05, ** *P*<0.01, and ****P*<0.001.

Supplementary Table S1. Primers used for cloning plasmids in this study.

| Primer Name | Primer Sequence (5' to 3') |
| --- | --- |
| shCTCF-F | CCGGGGAGAAACGAAGAAGAGTACTCGAGTACTCTTCTTCGTTTCTCCTTTTTG |
| shCTCF-R | AATTCAAAAAGGAGAAACGAAGAAGAGTACTCGAGTACTCTTCTTCGTTTCTCC |
| shNRF1-F | CCGGGAAACGGCCTCATGTATTTCTCGAGAAATACATGAGGCCGTTTCTTTTTG |
| shNRF1-R | AATTCAAAAAGAAACGGCCTCATGTATTTCTCGAGAAATACATGAGGCCGTTTC |
| shOGT-F | CCGGTTTAGCACTCTGGCAATTAAACTCGAGTTTAATTGCCAGAGTGCTAAATTTTTG |
| shOGT-R | AATTCAAAAATTTAGCACTCTGGCAATTAAACTCGAGTTTAATTGCCAGAGTGCTAAA |
| shBAP1#1F | CCGGCAACCGTGCTGTCCGTGATCTCGAGATCACGGACAGCACGGTTGTTTTTG |
| shBAP1#1R | AATTCAAAAACAACCGTGCTGTCCGTGATCTCGAGATCACGGACAGCACGGTTG |
| shBAP1#3F | CCGGGAGCAAAGGATATGCGATTCTCGAGAATCGCATATCCTTTGCTCTTTTTG |
| shBAP1#3R | AATTCAAAAAGAGCAAAGGATATGCGATTCTCGAGAATCGCATATCCTTTGCTC |
| shNTC-F | CCGGTGGTTTACATGTTTTCTGACTCGAGTCAGAAAACATGTAAACCATTTTTG |
| shNTC-R | AATTCAAAAATGGTTTACATGTTTTCTGACTCGAGTCAGAAAACATGTAAACCA |
| OGT-ATG-F | GGATCCATGGCGTCTTCCGTGGGCAACGTG |
| OGT-STOP-R | GGATCCTTATGCTGACTCAGTGACTTCAACAGGC |
| OGT-H911AF | CTCCTAAAGAGGAAGCCGTCAGGAGAGGCC |
| OGT-H911AR | GGCCTCTCCTGACGGCTTCCTCTTTAGGAG |
| NRF1-ATG-F | CCCAAGCTTATGGAGGAACACGGAGTGACCCAAACCG |
| NRF1-STOP-R | TTGGATCCTCACTGTTCCAATGTCACCACC |
| CTCF-ATG-F | CCCAAGCTTATGGAAGGTGATGCAGTCGAAGCCA |
| CTCF-STOP-R | GCGAATTCTCACCGGTCCATCATGCTGAG |
| RHOJ ATG-F | CCGGAAGCTTATGAACTGCAAAGAGGGAACTGAC |
| RHOJ STOP-R | AAGAATTCTCAGATAATTGAACAGCAGCTGTG |
| BAP1P-1k-F | GCTAGCAGCTTGTTGAGAAAATAAAAAATAAATAAACGTGTTCGACCCGCCG |
| BAP1P-1-HD3R | GGGAAGCTTACCAAGGCGCGACGGCCCTG |
| BAP1P-370F | CTCGCCCCACCTGCGCCCAG |
| BAP1P-350R | CTGGGCGCAGGTGGGGCGAG |
| BAP1P-46MutF | CGCATCTGCTGTCCGACAAAAAAAAGACGAGCCCAGAGGC |
| BAP1P-46MutR | GCCTCTGGGCTCGTCTTTTTTTTGTCGGACAGCAGATGCG |
| BAP1P-95MutF | CCGTCCCTCCGCAAGTAAGCGTTCGCCTTCGAGCGCATG |
| BAP1P-95MutR | CATGCGCTCGAAGGCGAACGCTTACTTGCGGAGGGACGG |
| OGTp-1k-F | GGTACCAATGTGACAAAATGTCTGAACAG |
| OGTp-1-R | AGATCTGGCGGCGACGGCTCCTCGGCCG |
| OGTP-1MutR | AGATCTGGCGTTTTTTGCTCCTTTTTTGAAACAACATGGGCCGGAAC |
| OGTp-191MF | CGGGTTGACAGGGCTGCAAAAAAAAAAGGTCTCCACCGTGGTGTTTGTCGC |
| OGTp-191MR | GCGACAAACACCACGGTGGAGACCTTTTTTTTTTGCAGCCCTGTCAACCCG |

Supplementary Table S2. Antibodies used in this study.

| Antibodies | Source | Catalog number (used concentration) |
| --- | --- | --- |
| BAP1 | Santa Cruz Biotechnology | sc-28383 (1:500 Western blot, 1:100 for immunohistochemistry) |
| CTCF | Santa Cruz Biotechnology | sc-271474 (1:1,000 Western blot) |
| NRF1 | Abcam | ab175932 (1;1,000 Western blot) |
| OGT | Proteintech | 11576-2-AP (1:2,000 Western blot) |
| RHOJ | Santa Cruz Biotechnology | sc-81936 (1:500 Western blot) |
| O-GlcNAc | Santa Cruz Biotechnology | sc-59623 (1:500 Western blot) |
| Lamin B1 | Santa Cruz Biotechnology | sc-374015 (1:500 Western blot) |
| α-Tubulin | Sigma-Aldrich | T9026 (1:2,000 Western blot) |


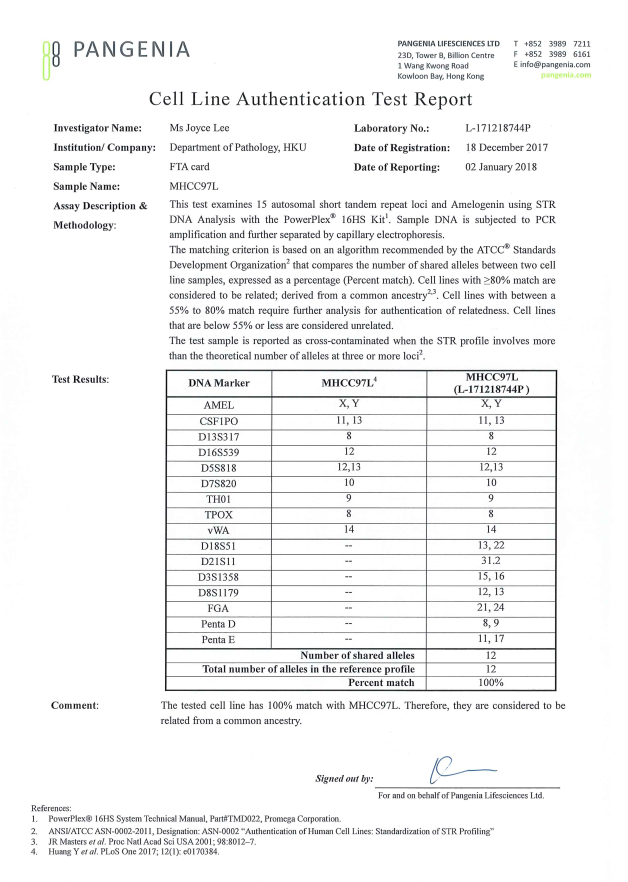


**Supplementary Fig. S3.** Short tandem repeat (STR) DNA profiling of MHCC97L.
