# Supplementary material for: Nuclear localization of BRCA1-associated protein 1 is important in suppressing hepatocellular carcinoma metastasis via CTCF and NRF1/OGT axis
Source: Cell Death Dis. 2025 Feb 21;16(1):123. doi: 10.1038/s41419-025-07451-0 (PMC11845619; doi:10.1038/s41419-025-07451-0)

Original Western blot

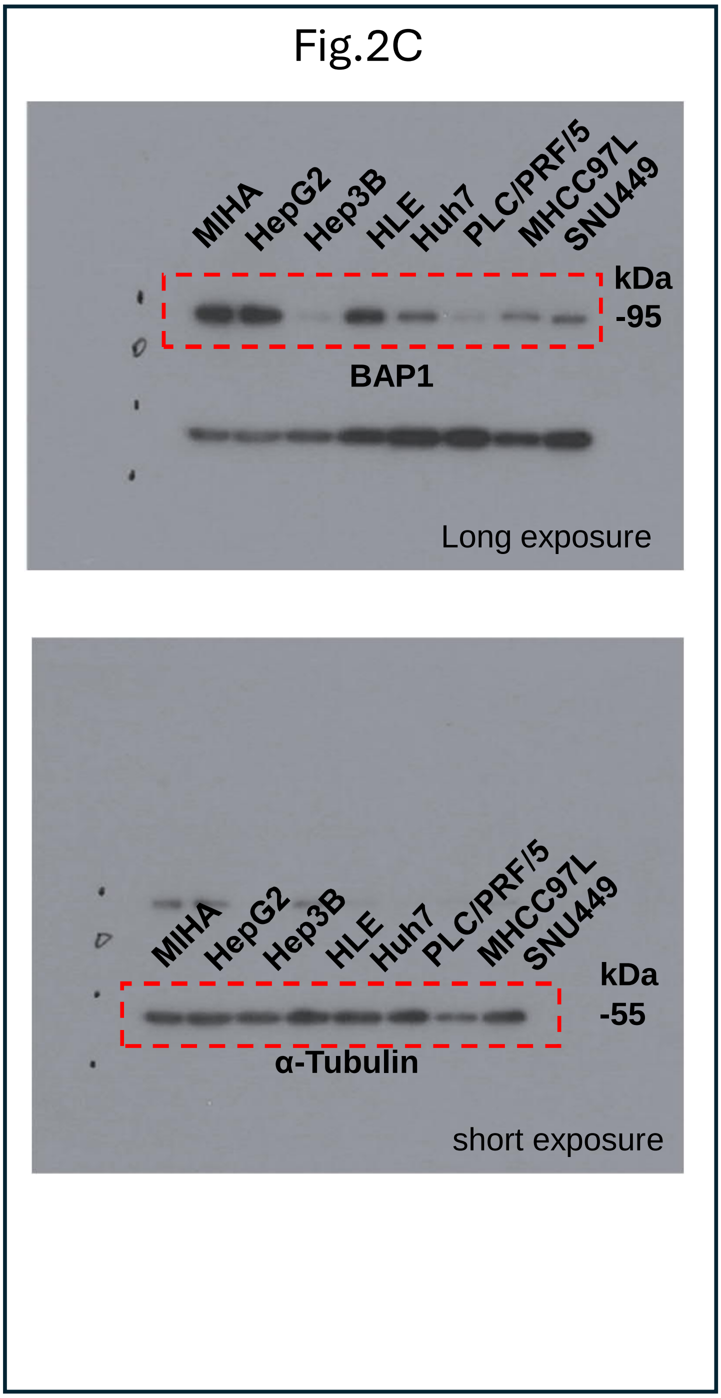

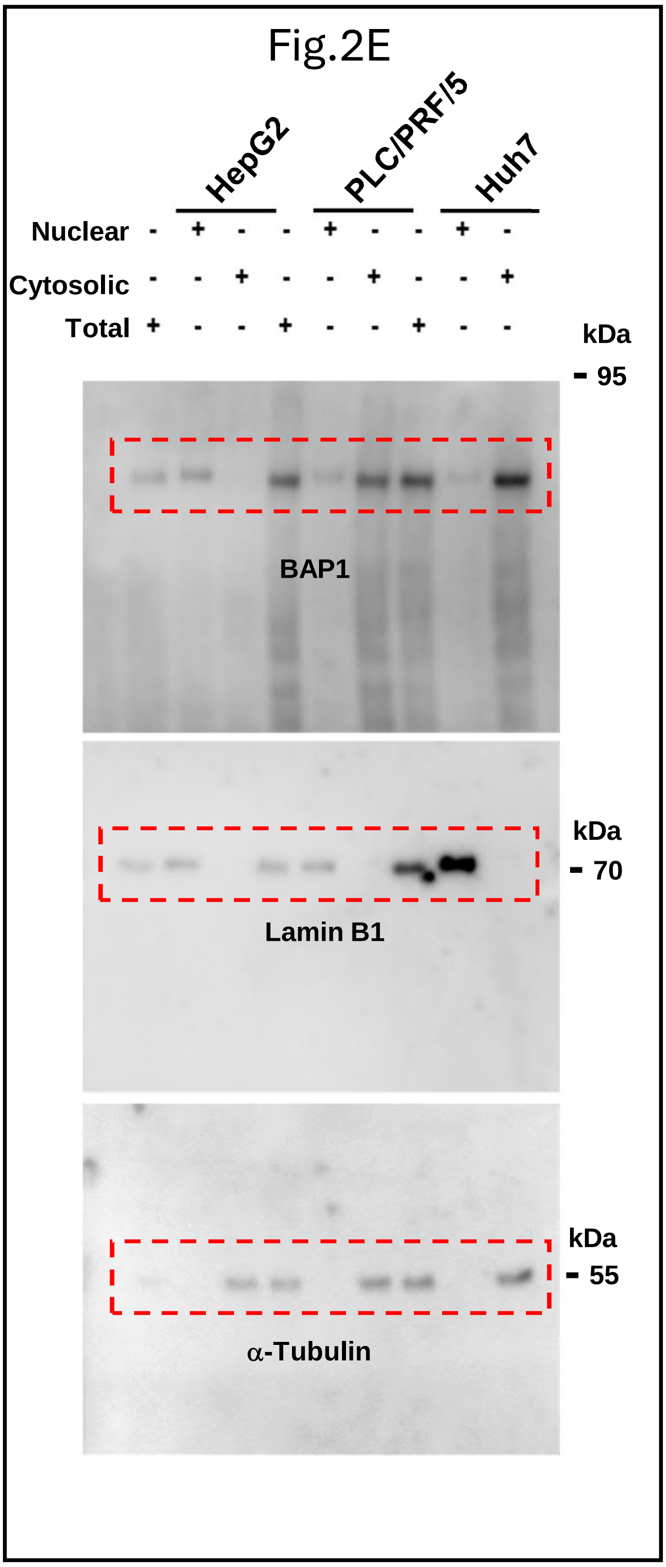

Fig.3A

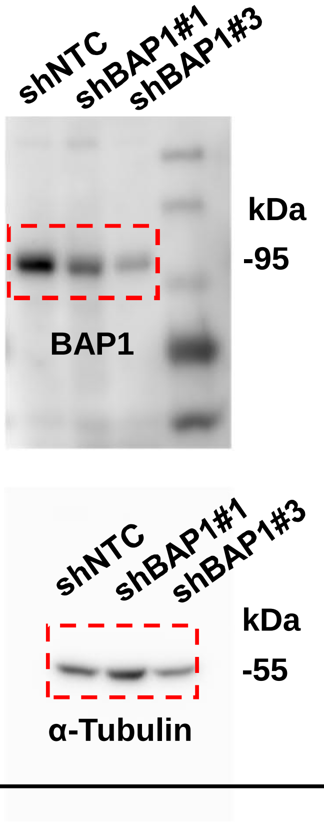

Fig. 4C

HepG2

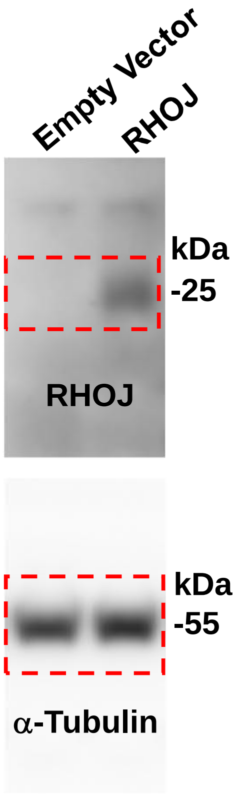

Fig. 4D

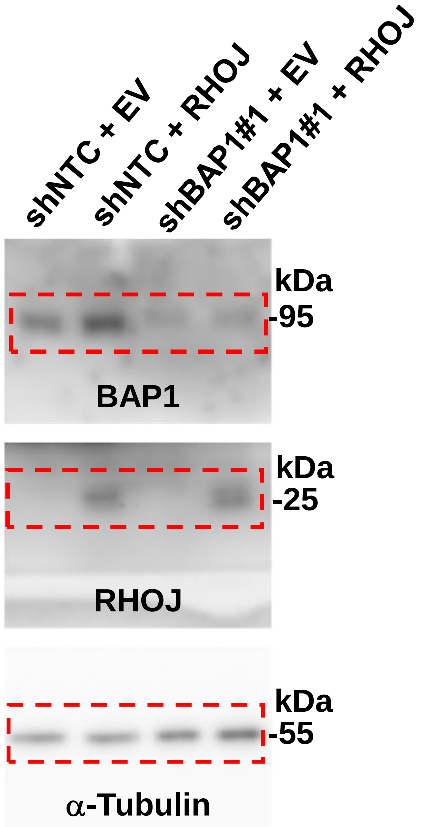

Fig. 5A (silver stain)

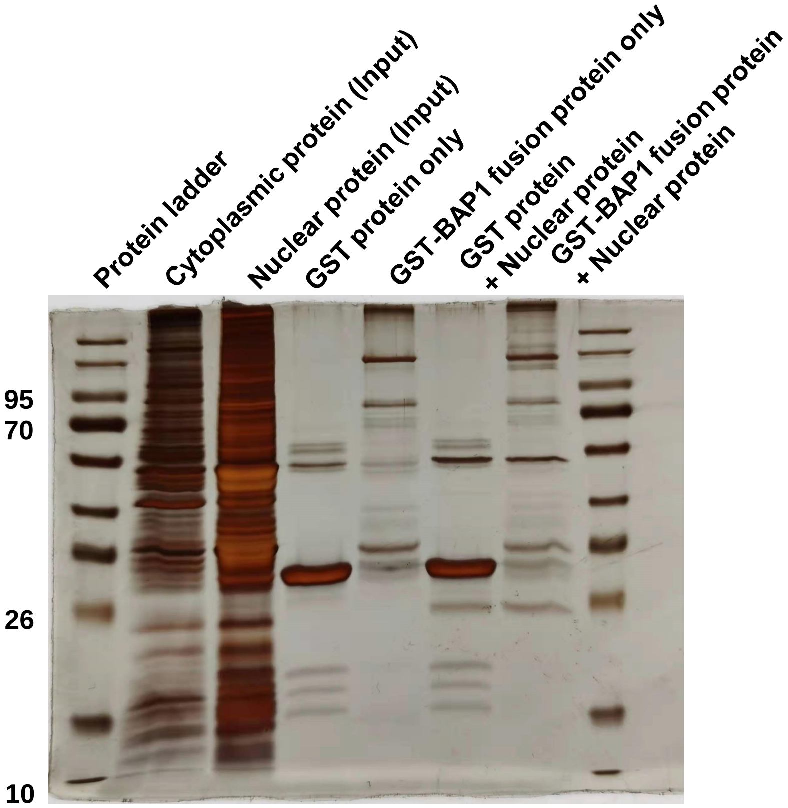

Fig. 5B

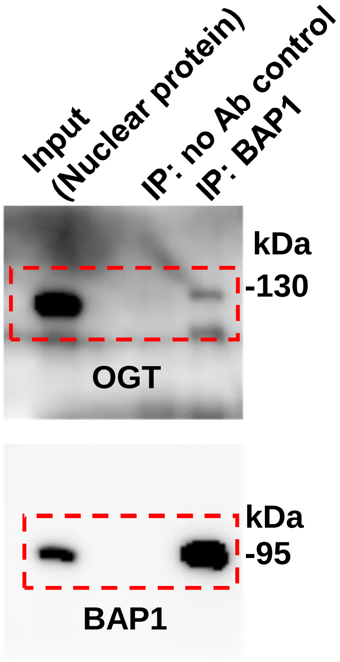

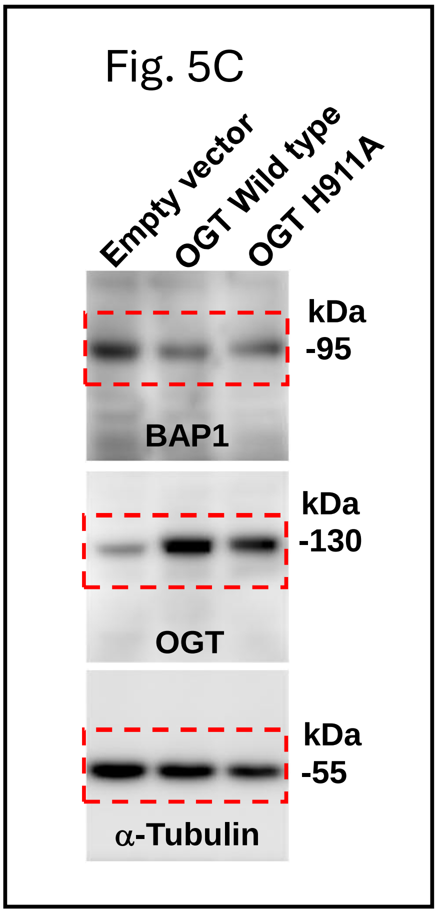
Fig. 5C
Empty vector
OGT Wild type
OGT H911A
kDa
-95
BAP1
kDa
-130
OGT
kDa
-55
α-Tubulin

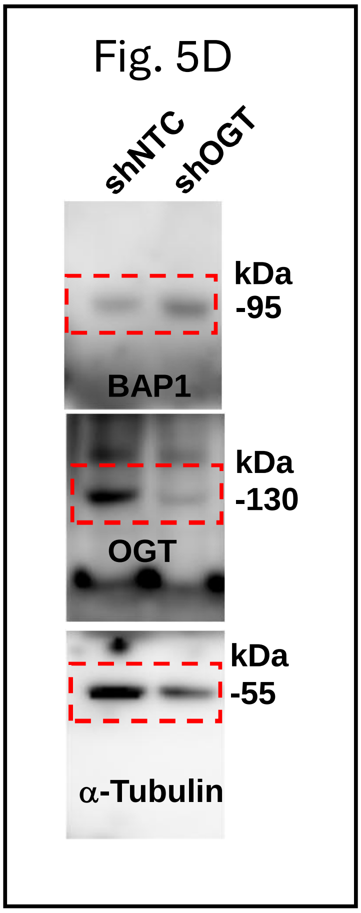
Fig. 5D
shNTC
shOGT
kDa
-95
BAP1
kDa
-130
OGT
kDa
-55
α-Tubulin

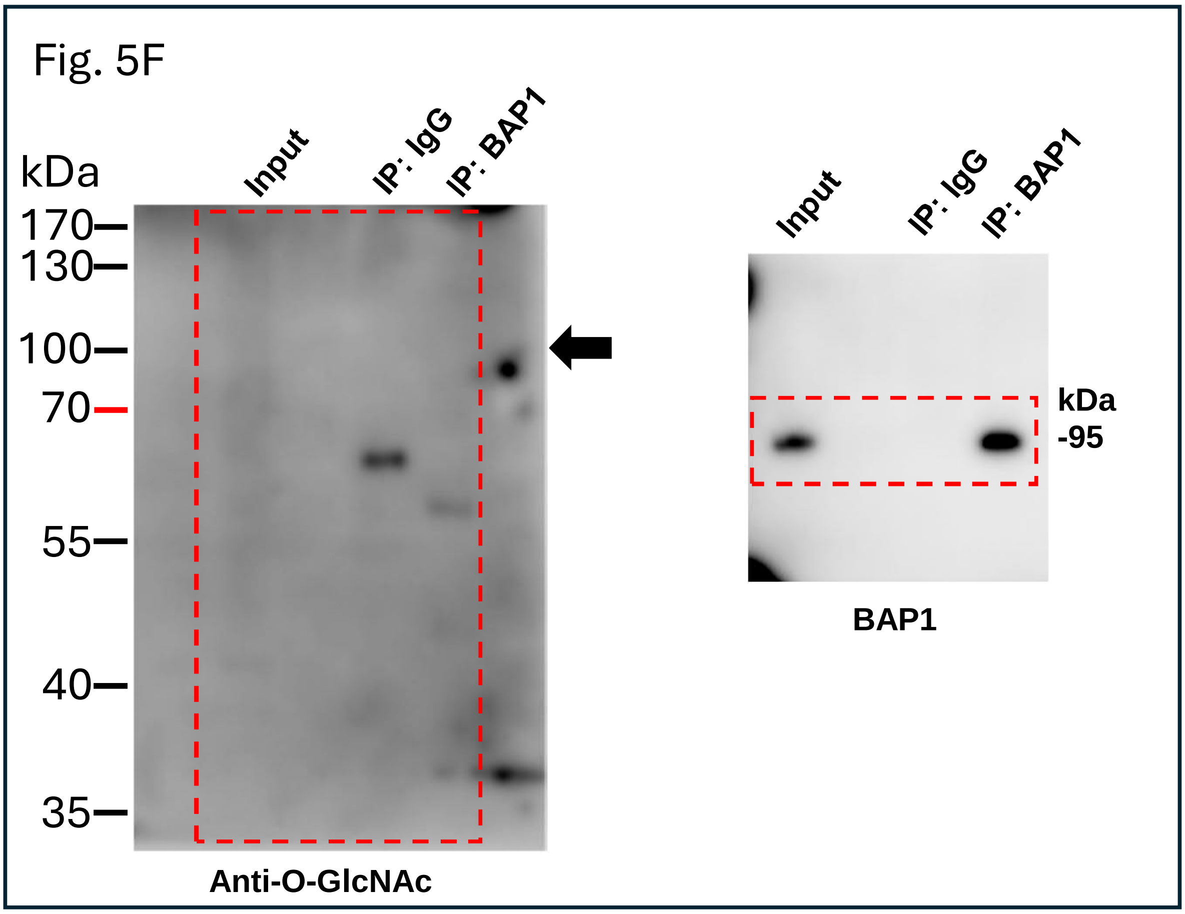
Fig. 5F
kDa
Input
IP: IgG
IP: BAP1
170
130
100
70
55
40
35
Anti-O-GlcNAc
Input
IP: IgG
IP: BAP1
kDa
-95
BAP1

Fig. 6D

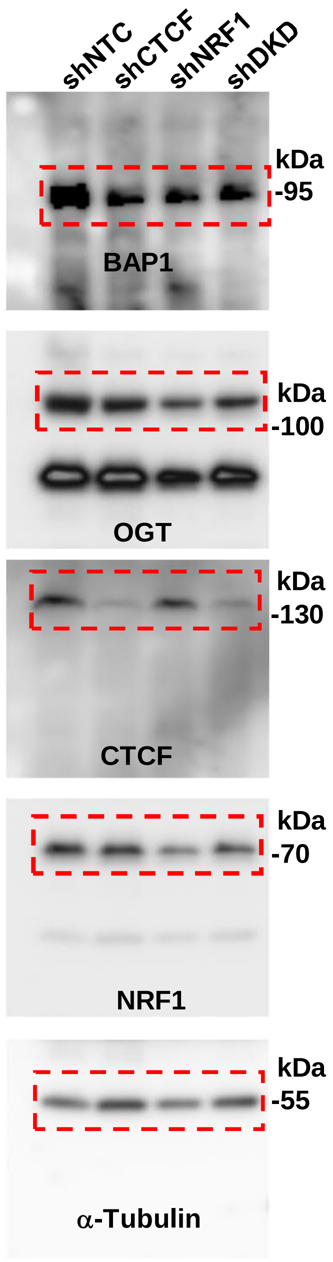
shNTC
shCTCF
shNRF1
shDKD
kDa
-95
BAP1
kDa
-100
OGT
kDa
-130
CTCF
kDa
-70
NRF1
kDa
-55
α-Tubulin

Supplementary Fig. S1

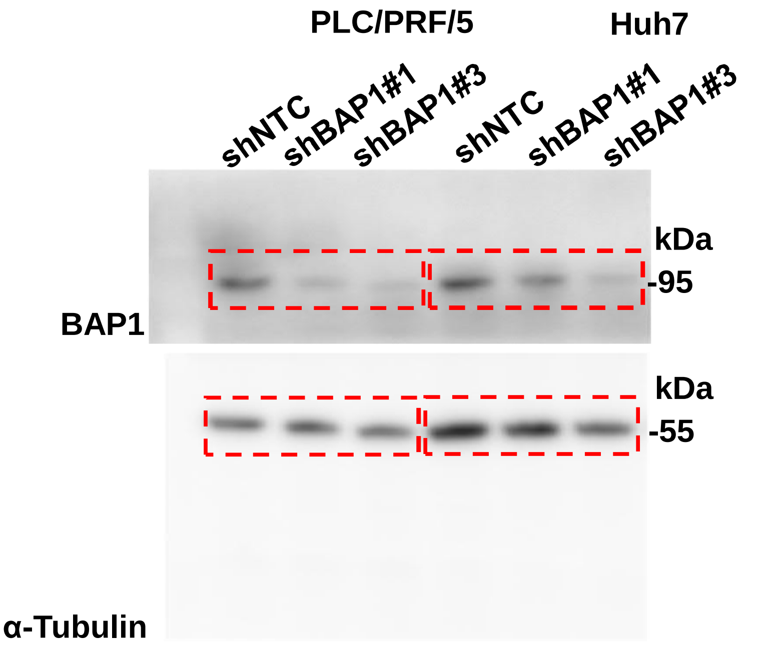

Supplementary Fig. S2

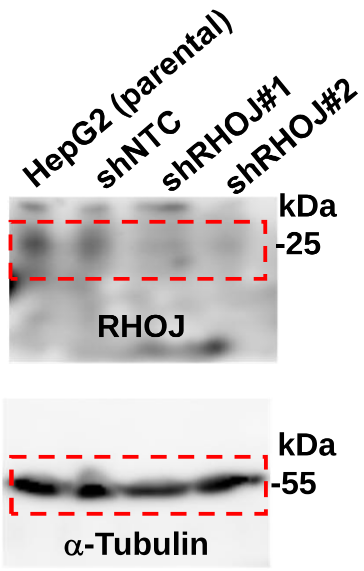

Supplement: Supplementary file 2 — Original Western blot [file 41419_2025_7451_MOESM2_ESM.pdf]
